# Review: Potential of Food Plants to Contribute to Human Intake of Per- and Polyfluoroalkyl Substances

**DOI:** 10.1021/acs.jafc.5c05889

**Published:** 2026-03-19

**Authors:** Adam H. Keith, Farzana Kastury, Albert L. Juhasz

**Affiliations:** Future Industries Institute, Adelaide University, Mawson Lakes Blvd, Mawson Lakes, Salisbury 5095, Australia

**Keywords:** PFAS, plant uptake, food contamination, human exposure, daily intake

## Abstract

Per- and polyfluoroalkyl substances (PFAS) are a class of anthropogenic chemicals that are ubiquitous in the environment. This review examined potential human exposure to PFAS through consumption of food plants, collating PFAS-plant bioaccumulation data, and highlighting the influence of different uptake factors. For commonly measured PFAS, significantly higher concentrations were reported in vegetative plants than in other edible portions. Estimated daily intake (EDI) values were calculated using minimum and maximum PFAS-plant bioaccumulation values and food consumption data for 3 countries. Food plants contributed the greatest EDI of any exposure route, accounting for up to 91.9% and 99.5% under maximum and minimum exposure conditions, respectively. In Australia and the US, wheat was the largest contributor to ∑PFAS maximum EDI from food plants, contributing 82.5–83.9%, due to the high consumption rate of wheat products. Due to regional differences in food consumption between northern and southern China, significant differences in PFAS EDIs were calculated.

## Graphical abstract

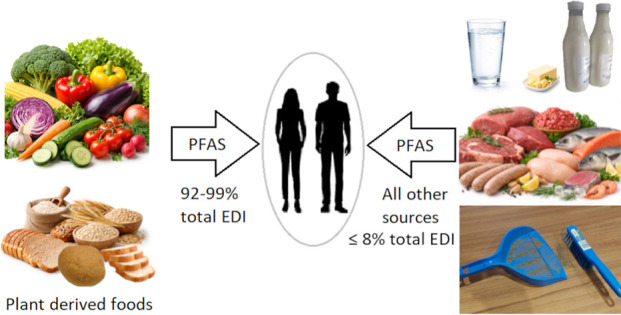

## Introduction

The group of chemicals known as per- and polyfluoroalkyl substances (PFAS) are synthetic organic compounds with carbon–hydrogen bonds substituted by carbon–fluorine bonds, with at least one fully fluorinated alkyl chain bonded to a functional group.^1^ This describes thousands of individual compounds, with more than 50 different structural categories,^2^ and a proportional number of chemistry related descriptors.^3^ Table S1 shows examples of some of the more common PFAS, the most investigated of which are perfluoroalkyl carboxylic acids (PFCAs) and perfluoroalkyl sulfonic acids (PFSAs). PFAS have received increasing amounts of interest in the past few decades due to their extensive use,^4^ ubiquitous presence in the environment,^5,6^ and related human and environmental health concerns.^7^ In particular, health and environmental concerns have led to three specific PFAS (perfluorooctanoic acid [PFOA], perfluorooctanesulfonic acid [PFOS] and perfluorohexanesulfonic acid [PFHxS]) being included on the Stockholm Convention’s persistent organic pollutants (POPs) list.^8^ Long-chain (8 ≤ C ≤ 20) perfluorocarboxylic acids, their salts, and related compounds have also recently been added to this list.^9^

The extensive use of PFAS stems from their unique physicochemical properties, including being hydrophobic and lipophobic,^4^ exhibiting versatile surface-active behavior,^10^ and having high chemical and thermal stability.^11^ These properties have led to the use of PFAS in many applications including: manufacture of consumer products, including electronics,^12^ textile stain-repellent treatment, metal electroplating, food packaging, and as an active ingredient in aqueous film-forming foams used for firefighting.^13^ However, due to the stability of carbon–fluorine bonds (485 kJ/mol), PFAS are highly resistant to degradation, including hydrolysis, metabolism, and photolysis.^14^ This has contributed to the persistence of these compounds in the environment and biota.^15^

The use of firefighting foams has been a significant source of localized environmental pollution from training exercises at airports and military bases.^16,17^ However, other sources that introduce PFAS into the environment include runoff and accidental leakages from facilities that manufacture or use PFAS^16,18^ and leaching from domestic waste landfill.^12,19^ Wastewater treatment plants are unable to remove all PFAS from the influent, resulting in PFAS being reintroduced into the environment through treated water reuse or biosolids used as soil amendments.^19,20^

The subsequent use of PFAS-contaminated water or biowaste in agriculture presents a source of exposure to humans through ingestion of plant foods^18,21^ or livestock that has accumulated PFAS through its feed or water.^22^ As such, food is considered a main intake source for PFAS in humans,^15,23^ although other exposure sources include drinking water,^24^ incidental ingestion of soil and dust (particularly in young children),^25^ inhalation of compounds in air,^26^ and dermal absorption or incidental ingestion of personal care products.^27^

Past research on plant uptake of PFAS has been split into several focus areas. These include assessing human health risk from edible plants^28–30^ and plant phytotoxicity.^31–33^ Studies of site-specific contamination^34,35^ have been quite common due to local governmental concerns, which has led to studies investigating temporal trends of PFAS contamination^36^ and phytoremediation potential of particular plant species.^37^ Insights into uptake patterns and mechanisms have been gathered through research on soil sorption and binding of PFAS,^38,39^ cocontamination of PFAS and other compounds/contaminants,^40^ and plant-assisted degradation to more stable/accumulative PFAS.^41,42^ Plant uptake of PFAS has also been reported in studies with a different focus, e.g., PFAS trophic transfer^43^ and changes to chlorophyll production, gene expression, and other biological impacts.^33,44^

Due to the many variable objectives and methodologies of studies measuring plant uptake of PFAS, comparing data from one study to the next is challenging. Previous reviews have reported on plant bioavailability, phytotoxicity,^45,46^ uptake factors,^47^ phytoremediation,^48^ and environmental impact^24^ to varying degrees. In contrast, this review investigates PFAS uptake by food plants since ingestion is a primary intake route for humans and uses plant concentrations to quantify potential intake for comparison against regulatory guidelines. It then compares PFAS intake from food plants to other food types and intake routes to indicate relative exposure from different sources and demonstrates the significant potential of food plants to contribute to human PFAS intake. While previous studies and reviews have reported some estimated intakes, it is believed that this is the first to systematically use agriculturally relevant plant concentration ranges to do so. By using all available concentrations for intake calculations, this review aims to identify food plants that have the greatest potential for human intake of PFAS and areas in which future research could help minimize human exposure.

### Information Sources

Research on PFAS uptake in plants has been continually increasing over the last few decades, as reflected in the number of studies reporting data on PFAS concentrations in plants (Figure S1). For this review, international peer reviewed primary research articles were searched using Scopus, Web of Science, and Google Scholar on February 15th, 2023, for any sources published up to this date. Keywords searched in the full-text included “PFAS AND plant OR food”, “(per- or poly-) fluorinated OR fluoroalkyl AND plant uptake”. Reference lists of review articles were also searched for relevant articles that may have been missed in the searches. After duplicates were removed, 1456 articles were screened to find studies that (i) reported on plant uptake of PFAS, (ii) were published in the English language, and (iii) recorded uptake values, not just observations of plant health/survival. Review articles and multiple articles reporting on different aspects of the same data were excluded from the list.

In the resulting 153 papers that reported PFAS plant uptake concentrations, 96 food plants and 106 other species were investigated, while three studies did not specify the grass or reed species used. More than 80% of papers investigated PFOS and/or PFOA, sometimes independently but often alongside other compounds. The other compounds assessed include mostly PFCAs and PFSAs or compounds that degrade into these two groups of PFAS. Plants were grown either hydroponically, in field collected soil, or in soil spiked with specific PFAS at concentrations ranging up to 350 mg/kg dry weight (d.w.).

### Factors Influencing PFAS Plant Uptake

The most investigated food plants assessed for PFAS uptake were lettuce (*Lactuca sativa*) and wheat (*Triticum aestivum*). Wheat is likely studied more because it is used extensively in flour and flour products, predominantly breads, and lettuce is studied because it is a common salad additive and grows relatively quickly. However, different plants have been shown to uptake PFAS at different rates based on a variety of factors, including plant species, growing conditions, and PFAS type. Moreover, different PFAS accumulate more in different plant compartments, further differentiating uptake between different plant types. The large variety of plants studied, along with different growing conditions, have resulted in many conclusions being specific to the study aims rather than reliably relating to the broader issue of PFAS in the environment.

Influence of plant type on PFAS uptake and translocation.

Directly comparing PFAS uptake using the concentrations in plants is not reliable since they have been shown to be dependent on PFAS concentrations in the growing media, among other factors. Figure S2 shows the range of reported PFAS concentrations in edible plant portions for food plants and media in which they were grown in. It can be observed that reported concentrations have varied by up to 8 orders of magnitude between studies. One method of addressing the wide range of concentrations investigated has been to relate PFAS concentrations in the plant portion to that of the growing media. For example, when PFAS-plant uptake is normalized by calculating bioaccumulation factors (BAF; the ratio of PFAS concentrations in the plant to concentrations in the growth medium; Figure 1), similar accumulation trends were observed for different food plant types, although BAF magnitude varied. Vine fruits such as tomato and cucumber accumulate shorter-chain (4–5 carbon) PFCAs (PFBA median BAF = 31.47, PFPeA median BAF = 17.1) by 2–4 orders of magnitude more than longer-chain (8–9 carbon) PFCAs (PFOA median BAF = 0.035, PFNA median BAF = 0.0126) and often do not accumulate detectable amounts of compounds with chain lengths ≳10 (Figure 1A).^30,49,50^ PFSAs show a similar reduction in BAF by compound length, albeit only by about 1 order of magnitude, between short-chain PFBS (4 carbon; median BAF = 0.42) and longer-chain PFOS (8 carbon; BAF = 0.045). This trend is repeated in vegetative food plants (Figure 1B), grains (Figure 1C), and root crops (Figure 1D). Specifically, studies involving particular vegetative plants, e.g., celery,^49^ lettuce,^50^ pak choi,^51^ red chicory,^52^ and spinach,^53^ have individually demonstrated this trend of decreasing PFAS accumulation for longer carbon chain compounds.

**Figure 1:**
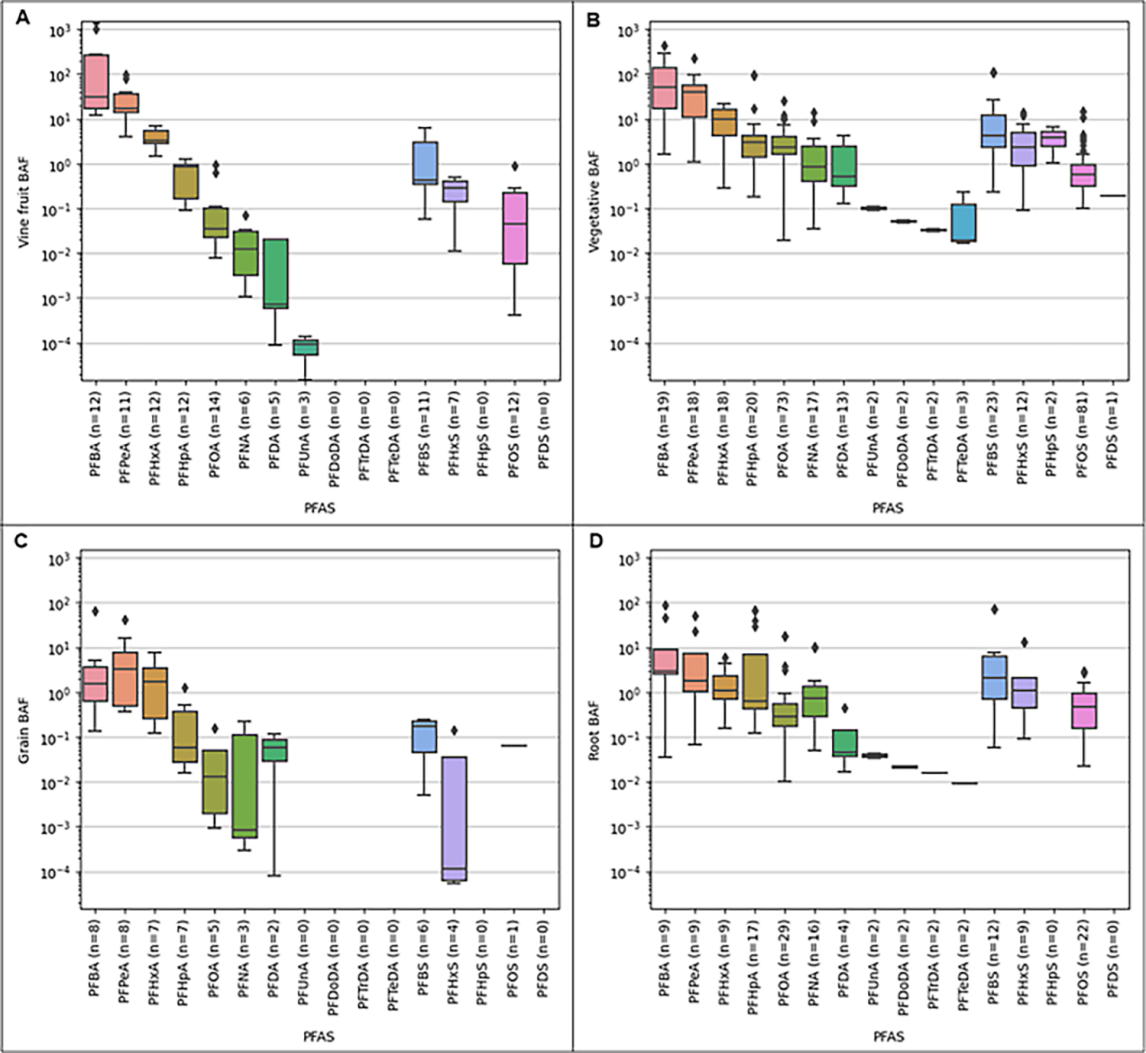
Boxplots of bioaccumulation factors (BAFs) for PFAS in the edible portion of (A) fruiting vines (cucumber, tomato, pea), (B) vegetative food plants (lettuce, celery, spinach, red chicory, alfalfa), (C) grains (wheat, maize), and (D) root vegetables (radish, carrot, potato) from the literature. Boxes are derived from 5 number summaries; diamonds show outliers beyond the 1.5× interquartile range. Nondetects are not included; multiple trials under different conditions reported in a single study are treated as separate data points.

Compared to vine fruits (median BAF 0.013–31.5) and particularly vegetative plants (median BAF 0.52–52), grains (median BAF 1.1 × 10^–4^ to 3.25) exhibit lower PFAS accumulation for compounds reported. Individual studies have not necessarily reflected this magnitude of difference; however, Wen et al.^54^ found that although wheat grains accumulated less PFAS than the straw, the BAF was still within an order of magnitude between the two. Additionally, the BAF (1.23–41.9; carbon chain length 4–8) of wheat shoots (grains and straw combined) investigated by Lan et al.^55^ aligned more closely with vegetative BAFs than that of grains, as shown in Figure 1. It is important to note that there were fewer BAFs reported for grains, which may contribute to its less clear trend in Figure 1C. Additionally, Yamazaki et al.^56^ demonstrated that the majority of PFAS in rice grains was in the hull and bran, which may be the case for other grains, and remains an area that warrants further research. This differential partitioning of PFAS within plant components presents a potential means of reducing human intake through food processing and preparation while demonstrating that there are multiple locations that PFAS can be restricted or accumulated within a plant during uptake.

Accumulation in root vegetables (Figure 1D) shows the least variation of all plant compartments. Although the trend of longer-chain PFAS having lower BAF is still present, the median values for compounds with carbon chain lengths less than 10 are within approximately 1 order of magnitude (PFBA median = 2.92, PFOA median = 0.28). The trend can be attributed, at least in part, to the higher mobility of shorter-chain PFAS in soil^57^ due to higher sorption with increasing chain length of the hydrophobic tail.^58^ However, PFAS translocation and accumulation in the plant is significantly influenced by carbon chain length as long chain molecules have their movement inhibited by the Casparian strip, cambium, and other cellular barriers.^59–61^ The sulfonate group of PFSAs is larger than the corresponding carboxylate of PFCAs, likely leading to lower accumulation of PFASs beyond the root; this effect is lessened for longer-chain PFSAs since the relative size of the headgroup is lower.^49^ Root concentration factors for PFCAs and PFSAs have been reported to exhibit a U-shape relationship, decreasing from 4 to 6–7 carbon atoms and then increasing with carbon chain length up to ≳10.^62–65^ This pattern is not observed in all studies or in root food plants overall, as shown in the root BAF graph in Figure 1D, and may be related to either the growing conditions or a species-specific effect. A consideration that does not appear to always be factored into BAFs of root food plants is PFAS binding to the exterior of the root. It has been shown that some PFAS have higher concentrations in the peel of these plants,^66^ which could elevate the reported BAF for these compounds.

Another measure of the relationship between the PFAS concentration in the roots and shoots of a plant is the transpiration stream concentration factor (TSCF). This can give an indication of the PFAS concentration expected in other parts of the plant for a given root concentration. The TSCF has been shown to be significantly negatively correlated with hydrophobicity of the PFAS compound and root lipid content,^58,67,68^ while a positive relationship has been demonstrated with specific proteins^45,68,69^ and water demand or transpiration flow during growth.^68,70^ Even within plant species, there is some natural variation in uptake due to different protein and lipid contents. Higher protein concentrations have been correlated with higher shoot BAF of PFAS in different subspecies of lettuce.^71^ This results in different variants or subspecies accumulating different concentrations of PFAS; however, this shows much less variation than between species. Less than an order of magnitude variation has been shown between subspecies under the same conditions,^71,72^ compared to different species regularly having greater than an order of magnitude difference in uptake factors.^50,73^

### Effect of Growing Conditions on Uptake

Despite the trends shown in BAF for different plant components, there is variability in reported PFAS BAF for a given plant, particularly when grown under different experimental conditions. Examples of this include average BAFs varying between 0.1 and 0.65 for PFOS in lettuce leaves^74^ or between 0.1 and 3.8 for PFOA in carrot.^73^ PFAS bioaccumulation derived from hydroponic studies may differ by up to 2 orders of magnitude compared to values reported for soil, dependent on carbon chain length, location in the plant, and site-specific soil characteristics.^50,52,75^ Since there is no soil to bind to, longer carbon chain compounds in hydroponic solutions are freely available to be absorbed by plant roots, resulting in higher root BAF. Accumulation and translocation for different plant compartments are consequently impacted, although the effect may vary depending on the compounds and plants involved. It has been proposed that competitive sorption/desorption in the roots and subsequent effect of translocation to other compartments is at least partly responsible.^50,52^ However, studies of this type are limited, and investigation into the specific mechanisms and interactions responsible does not appear to have been undertaken at the time of review.

It has also been demonstrated that root formation occurs differently in soil compared to hydroponics,^6,52^ and these differences in root structure also impact uptake and translocation of various PFAS. Roots tend to be finer and more heavily branched in soil,^52,76^ which may result in more long-chain PFAS being filtered out by the Casparian strip.^59^ Thus, although hydroponic studies are considered easier to conduct and have less complexity in results, they cannot fully predict soil uptake for a given plant. Additionally, at higher PFAS concentrations, root length and surface area can be reduced,^77^ further impacting plant uptake capability.

Soil pH plays a pivotal role in PFAS sorption to some soil compounds, which can influence PFAS availability in soil solution. Sorption has been shown to increase for PFAS as pH decreases^78,79^ due to changes in surface charge and/or hydrophobicity of soil surfaces. This increase in sorption is also higher for compounds with high p*K*_a_ (>2) than for those with lower p*K*_a_ (≤2).^79^ This relationship between pH and uptake is further complicated by protonation of the acids at low pH. Root uptake and membrane permeation occurs at a higher rate for neutral or more strongly protonated states of weak organic acids than for their corresponding anionic state.^64^ Soil pH also influences plant growth, with pH values closer to optimal for growth potentially exhibiting higher uptake. Wheat grown by Zhao et al.^80^ accumulated higher PFAS concentrations by a factor of more than 1.4 at pH 7 compared to pH 4 or 10. It was suggested that plant stress may be responsible for lower uptake, a reasonable conclusion given that environmental stresses can influence root water uptake^81^ and thus uptake of nutrients and other compounds in pore water.

Organic carbon (OC) content is known to impact plant uptake by sorbing PFAS to soil,^39,82^ particularly longer-chain compounds, thus reducing the available quantity in pore water and subsequently reducing plant uptake. Xiang et al.^83^ identified OC content as having the greatest importance to root BAF relative to other factors including pH, PFAS concentration, and protein content. Lasee et al.^82^ grew plants in sand, representing soil with no OC or other binding sites; BAFs were significantly higher, in some cases by an order of magnitude, than those reported for soils containing around 2–2.5% OC.

### Other Factors Influencing Plant Uptake

Co-contaminants including other PFAS and metal cations in the form of oxides or other complexes can influence plant uptake and translocation. PFOS and PFOA have shown greater translocation to shoots of soybean when exposed to a mixture of PFAS compounds relative to the individual compounds.^84,85^ Since the transport of PFAS through the plant is dependent on transpiration, this effect is suggested to be greater in more hydrophobic molecules, i.e., longer-chain PFAS and PFSAs.^85^ However, roots do not appear to show the same degree of synergistic accumulation, which is proposed to be a result of inhibition by lipids competing for binding sites on root proteins.^69^ Electrostatic interactions between soil constituents, particularly soil organic matter (SOM), and PFAS influence their sorption to soil and thus their availability for uptake.^58^ The presence of metal cations increases PFAS sorption to SOM, particularly intermediate-chain compounds (5–8 carbons).^78^ These electrostatic interactions are the dominant sorption effect for zwitterionic PFAS, which contain both positive and negative charges, and contribute to the impact of pH on the uptake of these compounds.^79^ However, anionic compounds are still subject to cation influence, as shown by the presence of Zn^2+^ increasing root accumulation of PFOA while decreasing that of GenX.^86^

Exposure time in contaminated media affects PFAS accumulation and BAF. Following exposure to contaminated media, PFAS-plant concentration increases rapidly during initial stages and then plateaus as equilibrium is reached,^61,65^ following a first-order sorption kinetic model.^65^ However, plant growth rate, or more specifically transpiration rate, also influences concentrations and BAF.^61,71^ Li et al.^31^ showed less uptake than Li et al.^87^ for the same concentrations of PFOA and with a longer growth time. The main difference between the two studies were that the lettuce samples in Li et al.^31^ were germinated for 10 days then exposed to PFOA for 28 days, whereas those in Li et al.^87^ were germinated for 10 days, cultivated in PFOA-free nutrient solution for 3 weeks, and then exposed to PFOA for 10 days, ending the experiment at a slightly later growth time. This demonstrates that greater uptake, at least in vegetative compartments, occurs during the later stage of plant growth, when a larger volume of water is being translocated within the plant and there is a larger, less restrictive vascular column.

As has been shown, factors impacting plant uptake are numerous, complex, and interconnected, meaning comparing studies is challenging and only generalizations are able to be made. This results in difficulty in assessing human health risk from food plants being grown in impacted soil due to the wildly varying BAFs calculated in studies thus far. Consequently, estimating PFAS intake from food has only been undertaken using food from specific locations and does not necessarily apply to other locations even if they have similar contamination concentrations. This is especially the case for home grown produce—more than half of Australian households grow some form of produce,^88^ of which more than half increased their growing activities as a result of the Covid-19 pandemic.^89^ Home soil PFAS concentrations are largely unknown, and compost or other soil conditioners may add PFAS to the soil in which produce is being grown,^90^ influencing soil-solution (or exchangeable) PFAS available for absorption.

### Estimating Human PFAS Intake

Apart from the environmental impact, one of the main concerns surrounding plant uptake is human PFAS exposure from the consumption of food plants. Recommendations of daily intake limits can range from 1000 ng/kg b.w./day for PFBA^91^ to 0.63 ng/kg b.w./day for PFOA, PFNA, PFHxS, and PFOS combined.^92^ These recommendations have been lowered considerably in the past decade, but only a handful of compounds have been studied sufficiently to have evidence of health impacts and therefore an indication of tolerable limits. Since food plants are a significant primary source of PFAS intake, estimating human intake is of vital importance.

Estimated human intake from food plants reported in previous studies.

Of the 110 food plant studies included in this review, only 18 calculated some form of PFAS intake estimate, while the remaining studies focused on aspects other than human health exposure. Only nine studies included PFAS intake estimates that were derived directly from food grown in field soil and used local consumption statistics to calculate an estimated daily intake (EDI) that could be referenced against heath authority recommendations. Table 1 shows a summary of EDIs calculated by the nine studies reviewed; more details are shown in Table S2. EDIs calculated in market basket studies by Heo et al.,^93^ Herzke et al.,^94^ and Klenow et al.^95^ have also been included in Table 1 for additional context. These were not included in the plant uptake studies reviewed since they were based on analysis of fresh produce sourced from supermarkets. Lasee et al.^73^ did not calculate an EDI but determined that soil concentrations of 9.7 μg/kg for PFOA and 90.5 μg/kg for PFOS would cause intake to reach reference doses of 20 and 30 ng/kg b.w./day, respectively, based on BAFs determined through their study.

**Table 1:** Estimated Daily Intakes of PFAS Compounds Calculated by Previous Studies Using Concentrations in Foods, Local Consumption Data, and Average Body Weight

Authors	Location	Plants studied	EDI from individual foods (ng/kg b.w./day)
^30^	China^a^	tomato, cucumber	<LOD – 2.62
 ^96^	China^a^	tomato, cucumber, eggplant, capsicum (pepper), Chinese cabbage	<LOD – 207^b^
 ^97^	Korea^a^	apricot, white cabbage, Chinese cabbage, green onion, parsley, lettuce, rice, plum, raspberry, spinach, tomato	<0.001–0.247
			Sum PFAS 0.144–0.530^b^
 ^63^	China^a^	lettuce, Chinese cabbage, chrysanthemum, cucumber	Approx. values^c^
			<LOD – 104
 ^93^	Korea	radish, onion, spring onion, beansprouts, spinach, kim chi, potato, sweet potato, cucumber, green pumpkin, cabbage	<LOD – 18.43
			Sum PFAS 4.23–86.97
 ^94^	Europe: Belgium, Czech Republic, Italy, Norway	carrot, onion, tomato, courgette, cucumber, aubergine, peppers, cauliflower, cabbage, broccoli, lettuce variants, spinach, chicory, asparagus, celery, fennel, mushrooms, potato, peas, beans	Approx. values ^c^
			0.027–0.185
 ^95^ Same concentration data as^94^ above	Europe: Belgium, Czech Republic, Italy, Norway	As above	<LOD – 1.137
 ^98^	China^a^	white melon, white gourd, okra, cucumber, amaranth, Chinese little greens, tomato, gourd, leek, loofah, eggplant, pumpkin, spinach, peach, watermelon, grape, pear, muskmelon, pitaya	<LOD – 26.3^b^
			Sum PFAS 15.9–39
 ^29^	China^a^	wheat, maize	<LOD – 1219
			Sum PFAS 0.54–1682
 ^60^	China^a^	wheat, corn, radish, carrot, Chinese cabbage, Chinese chives, pepper, welsh onion, cauliflower, pumpkin, celery, soybean, lettuce	<LOD – 2,743^b^
			Sum PFAS 11.5–3544
 ^99^	China^a^	cabbage (used as representative for all vegetable consumption)	<LOD – 15.05
			Sum PFAS 6.59–26.16
 ^35^	China^a^	cucumber, sponge gourd, tomato, eggplant, sweet pepper, balsam pear, zucchini, cabbage, spinach, rape, radish, carrot	<LOD – 0.543^b^
			Sum PFAS 0.31–0.633^b^

a Crops contaminated by the nearby industry or experimental design.

b EDI range given is the sum of foods investigated, not individual foods.

c Plant concentrations estimated from the graph since exact values were not provided.

Although EDIs might be below the current recommended quantities for several regulatory bodies, they only form part of the daily intake by humans. First, most of these studies only quantify a portion of the total dietary intake, making calculations based on part of the vegetable diet, which also does not include other known sources of PFAS such as seafood, meat, dairy products,^93^ and eggs.^96^ Additionally, there are other significant sources of PFAS in everyday life, including drinking water^27,100^ and indoor dust.^101,102^ Moreover, Australians in general (along with other Western countries) eat less fruit and vegetables than recommended; ∼95% do not meet the recommended daily intake of vegetables, and ∼74% do not meet the recommended intake of fruits.^103^ It is also evident that several studies utilized soil that exceeded human health investigation levels for residential soil, including 10 μg/kg for the sum of PFHxS and PFOS and 100 μg/kg for PFOA in Australia^104^ and 130 μg/kg for PFOS or PFOA in the United States.^105^ These Australian investigation levels are for residential soils based on the assumption that up to 10% of plant food intake is derived from home grown produce. The level should be proportionally lowered if the food plant intake is higher than 10% from that soil. This implies that agricultural soils should be approximately one-tenth of the values given here.

Determining estimated daily intakes from reported plant concentrations in the literature.

EDIs were calculated from studies conducted in soil for which plant concentrations were reported, with the inclusion of two market basket studies, to obtain a better understanding of total PFAS consumed through food plants. Source studies were limited to those with lower soil contamination, i.e., <100 μg/kg for individual PFAS. PFOA slightly exceeded this in a few cases but was included since other compounds were considerably lower than this threshold, and PFOA is often found in higher concentrations in soil than other compounds. This limit was chosen in an attempt to represent PFAS concentrations in typical agricultural soil, as opposed to more contaminated soil from industrial sites or phytotoxicity studies. This soil concentration also lies below the US regional screening level for PFAS in domestic soil, the lowest being 130 μg PFAS/kg.^105^ The range of intakes is presented in Table S4, where EDI was calculated using eq 1

1 $$EDI=\frac{S\times C}{W}$$

where EDI = estimated daily PFAS intake (ng/kg body weight/day), *S* = estimated daily intake of food per capita based on supply data (g/day) (Table S4), *C* = PFAS concentration in the edible portion of the plant (ng/g), and *W* = average body weight of an Australian adult (79.2 kg).^106^

The spread of total EDIs for PFCAs and PFSAs is shown in Figure 2 for those compounds that were detected in more than a single food source. Minimum values for each compound were calculated using the sum of the minimum EDIs for each food for which there were data; likewise, maximum values were the sum of maximum EDIs. The highest intake for Australians is PFBA, with a minimum EDI of 14.4 ng/kg b.w./day and a maximum of 880 ng/kg b.w./day. This predominantly originates from wheat consumption due to the high accumulation of PFBA in grains and the fact that Australians consume more wheat than any other plant-derived food. These estimates are limited to only food where data were available; it is likely that these values may be higher if calculations could accommodate data for all plant consumption. Shorter-chain PFAS—particularly PFBA—were present in food plants at higher concentrations overall. EDIs for PFCAs decreased as carbon chain length increased, with PFBA (14.4–880 ng/kg b.w./day) being consumed 2–3 orders of magnitude higher than PFUnDA (0.18–0.82 ng/kg b.w./day). This follows the trend of plant uptake decreasing with carbon chain length, with some variation that stems from the different number of studies investigating each compound and the variable concentrations in soils. PFSAs do not follow the same trend of EDIs; however, the maximum EDI for PFHxS was dominated by one wheat study, while PFOS had a greater number of foods contributing to the EDI and higher average soil concentrations than the other PFSAs.

**Figure 2:**
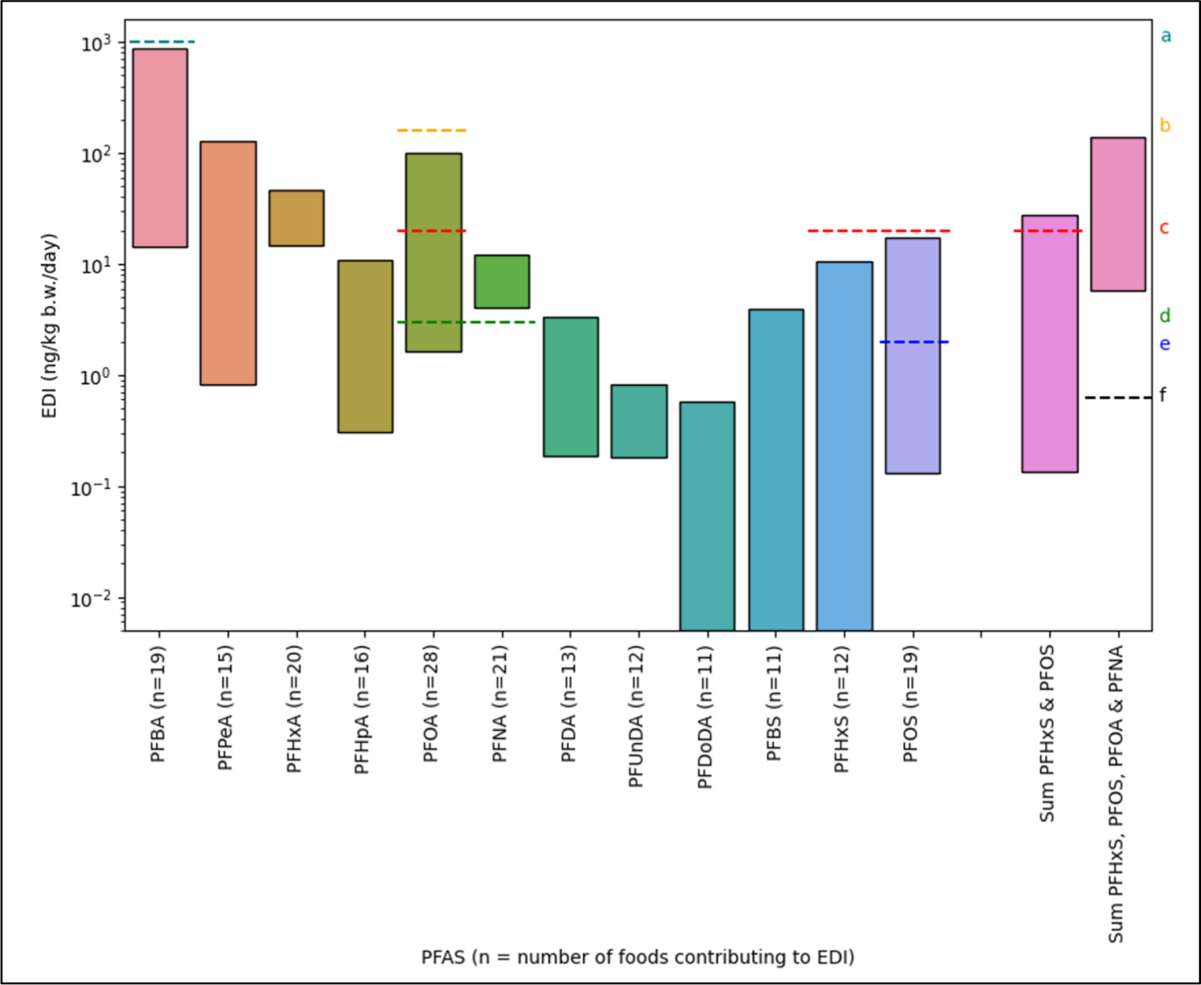
Range of total EDIs for Australian adults from food plants based on minimum and maximum reported plant concentrations in uptake studies with <100 μg/kg PFAS concentrations. PFDoDA, PFBS, and PFHxS were below the LOD in at least one instance for each food plant reported. (a) Reference dose for PFBA^91^ (1000 ng/kg b.w./day). (b) Tolerable Daily Intake (TDI) for PFOA^111^ (160 ng/kg b.w./day). (c) Reference dose for PFOA and PFOS,^112^ TDI of the sum of PFOS and PFHxS,^111^ and Minimum Risk Level (MRL) for PFHxS^113^ (20 ng/kg b.w./day). (d) MRL for PFOA and PFNA^113^ (3 ng/kg b.w./day). (e) MRL for PFOS^113^ (2 ng/kg b.w./day). (f) TDI of the sum of PFOA, PFNA, PFHxS, and PFOS^92^ (0.63 ng/kg b.w./day).

Apart from one study, PFDA intake was less than 1 ng/kg b.w./day, even under high soil concentrations (93.5 μg/kg).^49^ Moreover, even with PFDA soil concentrations of approximately 100 μg/kg, tomato or pea fruit consumption did not contribute to intake values. PFDoDA had low soil concentrations (<0.4 μg/kg in reviewed field studies) and was undetected in the edible portion of food plants, indicating that it does not contribute to intake under normal agricultural conditions. Likewise, PFUnDA had soil concentrations below 0.9 μg/kg and a maximum plant concentration of 0.6 μg/kg. This means that most EDIs calculated were 0.01 ng/kg b.w./day or below, making intake of significant quantities of PFUnDA through plant-based food unlikely, although tolerable intake values are yet to be established. PFDS plant concentrations were only reported in two studies under field conditions; it was not detected in the tomato fruit even with a soil concentration of 61.2 μg/kg, and only had an estimated intake of 0.16 ng/kg b.w./day due to its uptake in lettuce. PFHxS was undetected in plants grown in soil with <5 μg/kg and only appreciably contributed to intake through lettuce and predominantly wheat. Despite PFNA often being detected in soils and plants, most soil concentrations were below 1 ng/kg, and most plants did not appreciably contribute to the EDI. Those that did contribute to the EDI, particularly wheat, potato, and onion, contributed 59.2%, 17.2%, and 9.8%, respectively, resulting in a maximum EDI of 12.2 ng/kg b.w./day.

PFOA soil concentrations tended to be significantly higher than those of other PFAS detected. Despite the BAF of PFOA being less than those of some shorter-chain compounds, higher concentrations in soil led to EDIs being closer in magnitude to the EDIs of these compounds. This may have also been impacted by the larger number of PFOA studies and thus the number of foods in which it has been detected. However, as with PFNA, the intake was dominated by the influence of wheat. PFOS was not detected in any of the foods in the market basket study by Sznajder-Katarzynska et al.;^107^ however, most of the foods tested were fruits, which tend not to accumulate PFOS or other longer-chain PFAS.

Wheat was by far the largest contributor to the majority of maximum EDIs, as is evident in Figure 3, contributing 84.0% to the maximum of ∑PFAS and providing 98.2% contribution to PFHxS and 91.6% to PFBA. Lettuce and wheat are the greatest risk for PFAS intake overall based on EDI relative to soil concentration; lettuce also contributed up to 73.1% of the maximum intake for PFBS. Wheat is the greatest source of PFAS intake from the foods examined, not only due to the high plant uptake but also because of the large amount of wheat products consumed by Australians. Wheat grains also accumulate considerably more PFAS than rice or maize, which may be attributed to the higher protein content compared to other cereals.^108^ Although numerous papers have discussed the influence of protein on PFAS uptake and translocation, it is important to note that this effect appears to stem from limited studies on PFOS,^71^ PFOA,^69^ and *N*-EtFOSAA.^41^ This is combined with the knowledge that PFAS binds to proteins in animals and extrapolated to PFAS in plants in general. In addition, He et al.^109^ suggested that the influence on uptake and translocation may be protein- and lipid-specific. People from regions that consume rice instead of wheat as a staple, such as Southern China and Southeast Asia, would likely have a lower PFAS intake from their diet, especially shorter-chain compounds that have a greater propensity to accumulate in wheat.

**Figure 3:**
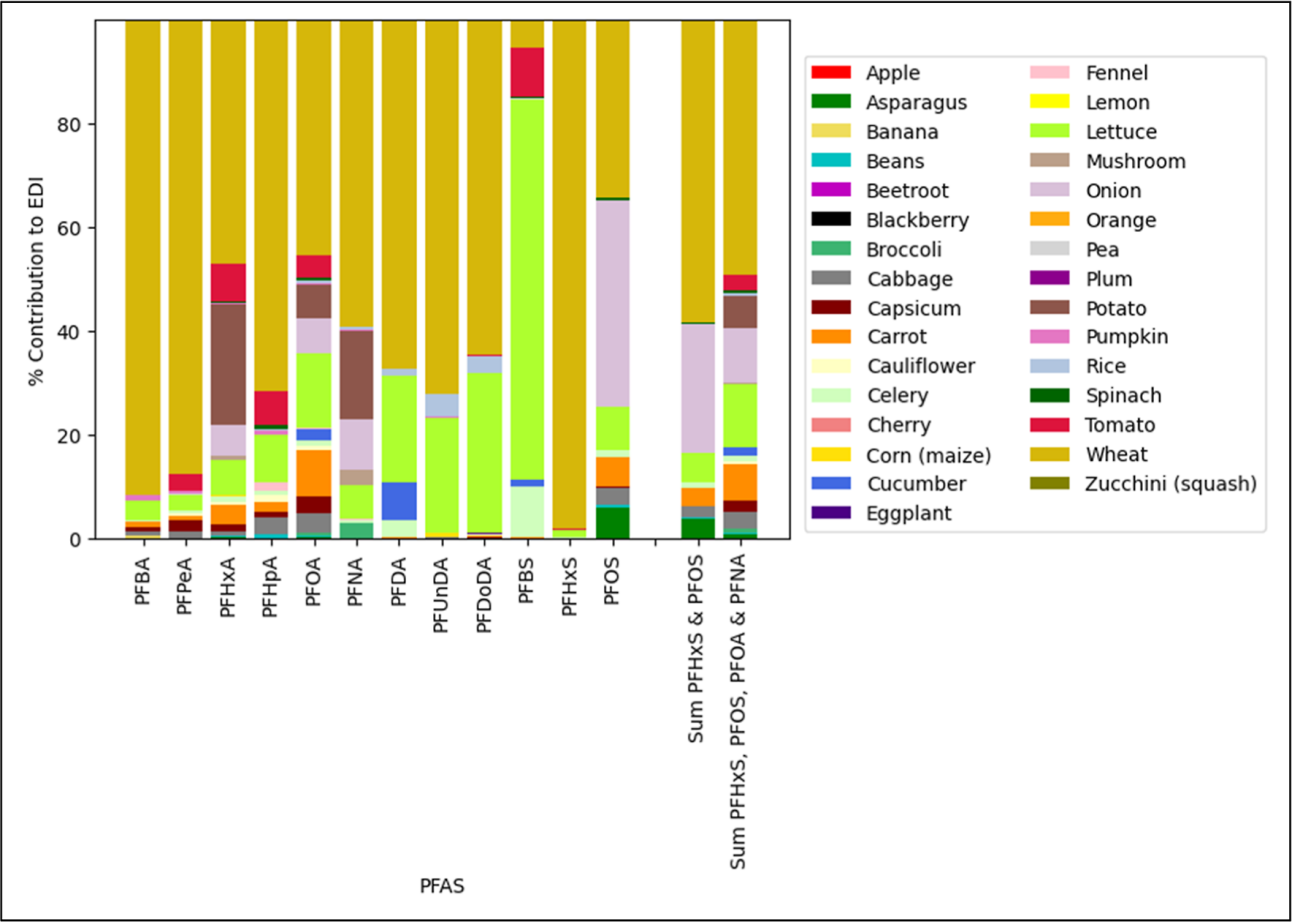
Percentage contribution of individual foods to the calculated maximum EDI for PFAS through consumption. Columns are stacked alphabetically by food from the bottom upward.

Tomato and other fruiting vines do not appear to accumulate longer-chain PFCAs (≥8 carbons) and especially PFSAs. Even in studies where PFOS and PFOA soil concentrations were an order of magnitude higher than those for other shorter-chain PFAS, their concentration in fruit was comparable to or lower than the concentration of those compounds. PFBA, PFPeA, and PFHxA accumulated in tomatoes, with concentrations in the fruit being higher than that of the soil they were grown in.^30,74,110^ With the exception of wheat, soil concentrations of approximately 1 μg/kg and below result in ingestion of less than 1 ng/kg b.w./day and often less than 0.01 ng/kg b.w./day for any given PFAS in any food plant.

Less investigated PFAS gave EDIs of

•0.18–1.19 for TFA in maize from Lan et al.^114^
•0.16 for PFTrDA in lettuce from Felizeter et al.^115^
•0.12 for PFTeDA in lettuce from Felizeter et al.^115^ and 0.13 in wheat from Wen et al.^54^
•<0.01–0.05 for PFHpS in lettuce and <0.01 in tomato from Blaine et al.^74^
•<0.01–0.16 for PFDS in lettuce from Blaine et al.^74^

As shown in Figure 2, the maximum EDI from the sum of 19 foods reported to contain PFBA is below the reference dose for lifetime exposure of 1000 ng/kg b.w./day given by the USEPA,^91^ although it is relatively close to this threshold and does not include intake from other foods or sources. Likewise, the maximum EDI (101 ng/kg b.w./day) for PFOA was lower than the tolerable daily intake (TDI) of 160 ng/kg b.w./day provided by Food Standards Australia & New Zealand.^111^ However, more conservative guidance is exceeded even at the minimum calculated EDI. While the maximum EDI calculated for PFOS was below the USEPA reference dose of 20 ng/kg b.w./day, the maximum EDI of PFOA (101 ng/kg b.w./day) was five times this value. Likewise, calculated PFHxS EDIs fell below the minimum risk level (MRL) of 20 ng/kg b.w./day,^113^ although the TDI of sum PFHxS and PFOS^111^ was exceeded by the maximum EDI. The maximum EDI for PFOA is nearly 34-fold higher than the MRL of 3 ng/kg b.w./day recommended by the Agency for Toxic Substances and Disease Registry,^113^ while the minimum EDI (1.64 ng/kg b.w./day) is over half of this limit. Due to the narrower range of EDIs, PFNA exceeds this same MRL even under minimal intake conditions (4.01 ng/kg b.w./day), despite fewer food plants being included in calculations.

The MRL of 3 ng/kg b.w./day for PFOA is far below the maximum EDI and less than double the minimum EDI (1.64 ng/kg b.w./day) calculated, while the same MRL for PFNA is exceeded by even the minimum EDI (4.01 ng/kg b.w./day). While the minimum EDI for PFOS (0.13 ng/kg b.w./day) falls below the MRL of 2 ng/kg b.w./day, the maximum intake (17.4 ng/kg b.w./day) is more than 8-fold higher than this limit. Most concerning is the TDI of 0.63 ng/kg b.w./day provided by the European Food Safety Authority (EFSA) Panel on Contaminants in the Food Chain for the sum of PFOA, PFNA, PFHxS, and PFOS.^92^ Each of these four compounds individually has a maximum EDI of above 0.63 ng/kg b.w./day, while PFOA and PFNA exceed this value even under minimum intake conditions.

Bao et al.^96^ calculated a significantly higher EDI from tomato than determined here as rural Chinese adults were reported to eat approximately 8 times as much tomato per day and weigh approximately 20 kg less than the average Australian. Liu et al.^29^ showed higher EDI, which was partially accounted for by the difference in average body weight between Chinese and Australians. Maximum concentrations for PFAS in Liu et al.^29^ were elevated for locations impacted by a fluorochemical plant. Uptake data for crops grown on-site or using downstream water for irrigation were removed when calculating EDIs in Table S3 as these do not represent typical exposure scenarios. Using crop concentrations from contaminated locations result in EDIs equivalent to those given by Liu et al.^29^ when adjusted for consumption and body weight differences between China and Australia (data not shown).

Observations from reported plant concentrations and EDIs have been made with the recognition that sample size is small (often one) for most data and soil concentrations mostly related to locations that were not heavily contaminated (mostly <5 μg/kg for individual compounds). Moreover, EDIs presented above only account for food plants where PFAS data have been reported, so data are lacking for not only other foods but also other exposure sources. In order to obtain a more comprehensive estimate of human intake of PFAS, other sources must be considered, including other food types such as seafood,^116^ meat,^22^ and dairy.^117^ Additionally, other exposure sources including drinking water,^24^ incidental ingestion of dust and soil,^102^ inhalation of volatile/particulate PFAS,^26^ and dermal absorption from personal care products, clothing etc.^27^ need to be accounted for.

### Comparing PFAS Intake from Plant Consumption to Other Exposure Routes

PFAS concentration data were collected from available studies for other food types. EDI ranges were calculated using eq 1 by replacing the plant concentration and daily consumption with the corresponding values for other foods (Table S5). Studies on PFAS concentrations in fish and seafood were the most common; data from four of these were collated for EDI calculations, providing a representation of different locations and seafood types. Data used for terrestrial animals were meat PFAS concentrations rather than the more readily available serum and organ concentrations. Most PFAS in poultry analyses have focused on liver and serum concentrations in addition to eggs; at the time of this review, no articles were found to report PFAS concentration in poultry meat. EDIs for dairy were calculated from consumption of three main foods consumed by Australians—milk, cheese, and yoghurt. Cheese and yoghurt PFAS concentrations were taken from Still et al.^118^ and Sznajder-Katarzyńska et al.,^119^ while milk values used a further two sources.^117,120^

Only one study was found to report PFAS concentrations in drinking water across Australia, and water concentrations from other studies were not used due to the significant variation of concentrations by location. EDI from drinking water was calculated using the recommended consumption of 2 L per day. Intake of PFAS from household dust occurs primarily from ingestion. Exposure due to inhalation of dust has been demonstrated to be negligible relative to other sources, Gustafsson et al.^101^ calculated the worst case EDI for the sum of 26 PFAS to be 7.7 pg/kg b.w./day for adults; thus, it has not been included in EDI calculations here. However, EDI from dust ingestion was calculated using eq 2, using a dust ingestion rate of 60 mg/day for adults^121^ and a gastrointestinal absorption value of 0.94 as given in de la Torre, Navarro, Sanz, and Martinez^122^

2 $$EDI=\frac{D\times C\times A}{W}$$

where EDI = estimated daily intake (ng/kg body weight/day) of PFAS, *D* = dust ingestion rate (0.06 g/day), *C* = PFAS concentration in household dust (ng/g), *A* = dust absorption factor (0.94), and *W* = average body weight of an Australian adult (79.2 kg).^106^

To determine intake through air inhalation, maximum and minimum air PFAS concentrations for individual compounds in Shoieb et al.^26^ were used to determine daily intake. These values were compared with EDIs determined by Poothong et al.^123^ to derive an overall air EDI range. Reliable methods of determining dermal absorption of PFAS from dust and cosmetics have yet to be developed, although modeling and in vitro experiments have indicated that dermal absorption accounts for <1% of human exposure.^124^ Including EDIs from Poothong et al.^123^ here likewise indicated that dermal absorption is negligible in comparison to other intake sources, although there is insufficient representation for this to be a definitive conclusion.

Table 2 and Figure S4 show EDIs for consumption and environmental sources. Food plants were the primary source of most PFAS for the total intake, comprising >90% of the total EDI under maximum and minimum exposure scenarios. The compound that showed the lowest intake from food plants, both in quantity and relative to other sources, was PFDoDA, contributing 6.2–7.3% of the total EDI. Seafood consumption had previously been shown to be a significant source of PFAS for human intake;^93,125^ however, the contribution to the overall maximum EDI calculated here was only 0.6%. The most significant intake from seafood comes from PFOA, PFUnDA, and PFOS, all of which contribute >2% to the EDI maximum; this result is consistent with previous intake estimates.^125^

**Table 2:** EDI of PFAS for Australian Adults from Dietary and Non-dietary Sources where Data Are Available

	EDI (ng/kg b.w./day)												
Source	PFBA	PFPeA	PFHxA	PFHpA	PFOA	PFNA	PFDA	PFUnDA	PFDoDA	PFBS	PFHxS	PFOS	References
Food plants	14.4–880	0.81–126	14.7–46.3	0.30–11.0	1.64–101	4.01–12.2	0.19–3.34	<0.01–0.82	<0.01–0.62	<0.01–3.94	<0.01–10.5	0.13–17.4	
Fish/seafood	<0.01–0.18	<0.01–0.38	<0.01–0.15	<0.01–0.06	<0.01–2.97	<0.01–0.35	<0.01–0.17	<0.01–0.63	<0.01–0.15	<0.01–0.01	<0.01–0.11	<0.01–2.82	^116,127,130^
Red meat	<0.01	<0.01	<0.01	<0.01–0.03	<0.01–0.05	<0.01	<0.01	<0.01–0.03	<0.01	<0.01	<0.01	<0.01–0.04	^120,128^
Poultry													None found
Eggs			<0.05	<0.11	<0.05–0.58	<0.05–0.43	<0.11–1.72	<0.11–0.97		<0.11	<0.05–1.12		^131^
Dairy	0.02–3.99	0.02–0.59	0.02–0.12	0.02–0.86	<0.01–22.4	<0.01–7.67	<0.01–10.3	<0.01–6.46	<0.01–9.16	<0.01–0.12	0.01–0.27	<0.01–20.6	^117,119,120^
Water			<0.01–0.14	<0.01–0.06	<0.01–0.24	<0.02	<0.02	<0.01		<0.01–0.06	<0.01–0.36	<0.01–0.39	^132^
Dust ingestion	<0.01	<0.01	<0.01–0.03	<0.01–0.05	<0.01–0.11	<0.01–0.04	<0.01–0.03	<0.01–0.04	<0.01–0.03	<0.01	<0.01–0.29	<0.01–0.48	^102,122^
Inhalation			<0.01–0.10	<0.01–0.03	0.01–1.25	<0.01–1.05	<0.01–0.47	<0.01–0.04	<0.01–0.13			<0.01	^26,123^
Dermal			<0.01	<0.01	<0.01–0.02	<0.01					<0.01–0.01	<0.01	^123^
Total	14.4–885	0.84–128	14.7–46.9	0.33–12.1	1.67–129	4.03–21.7	0.21–16.1	<0.01–9.0	<0.01–10.1	<0.01–4.15	0.02–12.7	0.14–47.1	

Red meat did not contribute significantly to the PFAS EDI, contributing less than any other source. However, concentrations used in calculations included only a limited amount of data (beef); other data for red meat were not found at the time of this review. In addition to red meat, PFAS may bioaccumulate in other compartments (e.g., offal),^126^ which may form part of the diet in some circumstances. PFOS has been shown to accumulate in the liver at concentrations up to 60-fold higher than in muscle tissue,^120,127,128^ which may significantly impact EDI calculations depending on consumption rate.

Eggs showed high concentrations of PFDA, PFUnDA, and PFOS, contributing 10.7%, 39%, and 11.1% to the maximum EDI respectively. Maximum intakes for longer-chain (C ≥ 8) compounds had significantly higher contributions from dairy (17.4–90.8%), mostly from consumption of milk. There was some variation in reported PFAS concentrations in dairy products; Hill et al.^129^ reported < MDL (1.6–144 ng/L) for PFCAs and PFSAs tested, although precursors were present, which have the potential to transform into these terminal degradant products. Macheka et al.^117^ reported significantly higher concentrations of C_9_–C_14_ PFCAs in dairy milk compared to other studies, which are reflected in maximum EDI calculations.

Drinking water had a low contribution to PFAS intake, providing 0.10–0.17% of the total EDI for the ∑PFAS calculated here, although it contributed 2.8% of the maximum for PFHxS. The current Australian Drinking Water Standards have guideline limits of 70 ng/L for the sum of PFHxS and PFOS and 540 ng/L for PFOA.^133^ The maximum concentrations reported in Thompson et al.^132^ are less than half for the sum of PFHxS and PFOS and 50-fold lower for PFOA relative to these guidelines. Accommodating these compounds at their regulatory limit would increase the maximum intake of sum PFHxS and PFOS to 1.8 ng/kg b.w./day, which would increase the contribution of drinking water to 2.9% of the total EDI for these compounds. This is a small contribution relative to other intake sources but would still exceed the TDI of 0.63 ng/kg b.w./day recommended by the EFSA. Increasing PFOA to its limit would have a more significant impact, adding 13.6 ng/kg b.w./day and increasing the contribution of drinking water from 0.19% to 9.6% of the total EDI maximum. However, the National Health and Medical Research Council is currently considering updating drinking water guidelines, reducing maximum allowable concentrations of PFOA, PFHxS, and PFOS to 200, 30, and 4 ng/L respectively. Consumption of drinking water containing PFAS at the proposed maximum concentrations would change contributions to EDI to 0.85 ng/kg b.w./day for the sum of PFHxS and PFOS and 5 ng/kg b.w./day for PFOA. This would considerably reduce the impact of drinking water to EDI for these compounds. Inhalation of PFCAs and PFSAs have a negligible contribution to the total EDI of these compounds (<0.4%), although volatile precursors may be present at higher concentrations, as reported by refs 26 and 134 . This may impact EDI calculations for the inhalation pathway, although this is also true of precursor influences for other exposure sources and pathways. As noted previously, dermal absorption has negligible contribution to PFAS intake, although data only accounted for absorption from household dust and not the potential impact of cosmetics where PFAS concentrations up to 19 mg/kg have been reported.^135^

It is important to note that regional differences exist that impact PFAS concentrations in drinking water and foods. Exposure from drinking water is particularly dependent on the water source and potentially has the greatest variability based on location. Other exposure studies for dust, air, and dermal absorption likewise focus on specific locations or conditions, which are not necessarily representative of the general populace. As such, there are limitations to using data from a small number of studies to calculate the EDIs. The purpose here is to give an indication of the other potential human PFAS exposure sources rather than a comprehensive meta-analysis of all exposure sources. Maximum EDIs presented here represent the worst case scenarios outside of sites significantly impacted by local contamination sources (e.g., water downstream from fluorochemical plants). Maximum calculated EDIs are also diet- and lifestyle-dependent; replacing animal derived foods with plants decreases the maximum available intake of compounds with a carbon chain length of ≥8 but increases potential intake of PFBA, PFPeA, and PFHxA by approximately 15%. Menzel et al.^136^ determined that individuals adopting a vegan diet had lower serum PFOS and PFNA concentrations, potentially related to the higher affinity of these PFAS to animal-based proteins. Greater consumption of seafood has also been linked to higher PFAS serum concentrations,^125,137^ although this is dependent on seafood type (pelagic versus estuary species; filter feeders versus herbivores, carnivores and omnivores), which influences PFAS concentration.

### Intake Variation Due to Differences in Diet

Due to regional differences in food consumption and exposure, local food consumption data (Table S6) and drinking water PFAS concentrations^138^ were used to calculate PFAS EDIs for the United States (Tables S7 and S8, Figure S5). The maximum PFAS EDI from food plants is comparable between the US and Australia (1.6% difference) due to the similarities in the diet. Calculated values highlight that US residents have lower PFAS intake from dairy but higher PFAS intake from seafood and drinking water. Higher maximum PFAS intake from US drinking water (6.7 ng/kg b.w./day for sum PFAS) contributed approximately five times more to the EDI compared to Australia. However, food plants still accounted for the majority of PFAS intake in the US, contributing 94.7–99.5% of the total 53.7–1298 ng/kg b.w./day for the sum of PFAS calculated here.

EDIs for China were also calculated using local city drinking water concentrations;^139^ however, consumption data for individual foods were unable to be sourced. By grouping foods into fruits, vegetables, rice, wheat, and other grains, estimated consumption (Table S5) and geometric mean of concentrations for all foods in each group were used to calculate EDI (Table S9). Geometric mean was used instead of average concentration since PFAS concentrations are not normally distributed. In addition, as Chinese rice and wheat consumption varies between northern and southern regions, EDIs were calculated to determine the impact of dietary influences on PFAS exposure. Calculated EDI for Chinese residents in northern regions highlighted higher intake for all PFAS (0.03–95.2 ng/kg b.w./day) compared to Australian and US populations due to higher consumption of vegetables, wheat, and rice (Table S10), which contributed up to 92% of the EDI. In contrast, calculated EDIs for Southern Chinese regions were significantly lower for PFBA, PFPEA, PFHxA, and PFHxS (by 58–70%) than for northern regions. These compounds along with PFBS have not been reported in rice (compared to wheat), which is the main grain consumed in Southern China. Although it is difficult to compare the contribution of food plants to total PFAS EDI (due to the method of calculation), the maximum PFAS EDIs for northern and southern China are 1.6-fold and 0.66-fold of values calculated for Australian diets using this same method. This illustrates the impact of diet, in particular wheat consumption, on PFAS EDI.

The contribution of water to PFAS EDI is more significant in southern regions (maximum of 32.0 ng/kg b.w./day for ∑PFAS), accounting for 19.9% of the maximum EDI, compared to northern regions (maximum of 2.98 ng/kg b.w./day for ∑PFAS), for which it contributes only 1.4%. This suggests that water monitoring and quality control are more relevant to managing human health risk in Southern China, particularly since irrigation water also contributes to PFAS in food plants.

## Conclusions and Future Research

This review highlights the potentially significant contribution of food plant consumption to the PFAS EDI. In some cases, calculated EDIs for compounds of concern exceeded recommendations by regulatory authorities. However, a constraint of these calculations was data limitations for PFAS concentrations in food plants. In many cases, only a small number of PFAS concentrations or a limited number of compounds were quantified, or in some cases, only average values were reported. PFAS concentrations in food also vary significantly, depending on local soil concentrations and contamination sources. Due to the limited data available, PFAS concentrations used to calculate EDIs were often not local, thereby not necessarily being applicable to the population for which the EDIs were calculated for. For many foods, data on PFAS concentrations were not available, which may result in an underestimation of calculated EDIs. Conversely, utilizing PFAS concentration data from studies where plants were grown in soil and/or irrigated with water artificially impacted with PFAS may overestimate PFAS concentrations typically found in plant foods. Expanding market basket surveys would overcome these limitations by providing PFAS concentration ranges for plant foods that are available to the public.

EDI calculations assumed that following consumption, all PFAS in food plants were 100% bioavailable, i.e. PFAS were released from the plant matrix in the gastrointestinal tract following ingestion and 100% absorption into the systemic circulation occurred. This conservative assumption does not consider the influence of physiological and physicochemical parameters on PFAS desorption and absorption, although the magnitudes of these influences on PFAS exposure are yet to be elucidated.

An omission in calculations that may influence EDI estimates is the impact of precursor compounds on PFAS exposure magnitude. A significant knowledge gap for food plants is the extent of precursor accumulation in edible portions and the propensity for transformation to terminal PFCAs or PFSAs following consumption. Conceivably, presystemic metabolism of desorbed precursors in the gut lumen, facilitated by the microbiome or liver cytochrome P450 enzymes, may result in higher-than-expected exposures for PFCAs or PFSAs. However, given the complexity of precursor analysis and the lack of relevant data, the impact of EDI calculations is unclear. To overcome this data gap, future studies should investigate the extent of precursor uptake into edible plant portions and their ability to desorb under gastrointestinal conditions. In addition, an assessment of precursor permeability and transformation susceptibility (in the gut lumen or liver) is warranted to determine the impact of in vivo processes on precursor transformation and exposure.

## Supplementary Material



## Notes

PFAS abbreviations and classes, EDIs for individual foods and food groups, consumption data, PFAS plant study chart, PFAS concentrations in soil and hydroponics graphic, combined BAF graphic, PFAS source percentage contribution graphic, and EDI for US adults graphic (PDF)
