# Supplementary material for: Review: Potential of Food Plants to Contribute to Human Intake of Per- and Polyfluoroalkyl Substances
Source: J Agric Food Chem. 2026 Mar 19;74(12):9857–72. doi: 10.1021/acs.jafc.5c05889 (PMC13047675; doi:10.1021/acs.jafc.5c05889)
Supplement: Supplementary file 1 [file jf5c05889_si_001.pdf]

Supplementary information for:

Review: Potential of food plants to contribute to human intake of per- and polyfluoroalkyl substances

Adam H. Keith[a*], Farzana Kastury[a] and Albert L. Juhasz[a]

[a]Future Industries Institute, Adelaide University, Mawson Lakes Blvd, Mawson Lakes SA 5095, Australia

*Email: adam.keith@adelaide.edu.au

Table S1 – Common PFAS classes, abbreviations, examples [1] and occurrence in reviewed studies reporting plant uptake of PFAS

PFAS group	Abbreviation	Examples	Occurrence in 153 reviewed studies
Perfluoroalkyl carboxylates	PFCA	Perfluorobutanoic acid (PFBA)<br>Perfluorohexanoic acid (PFHxA)<br>Perfluorooctanoic acid (PFOA)	71<br>79<br>125
Perfluoroalkyl sulfonates	PFSA	Perfluorobutane sulfonic acid (PFBS)<br>Perfluorohexane sulfonic acid (PFHxS)<br>Perfluorooctane sulfonic acid (PFOS)	75<br>71<br>122
Fluorotelemer alcohols	FTOH	6:2 fluorotelomer alcohol (6:2 FTOH)<br>8:2 fluorotelomer alcohol (8:2 FTOH)	4<br>5
Fluorotelomer carboxylic acids	FTCA	6:2 fluorotelomer carboxylic acid (6:2 FTCA)<br>8:2 fluorotelomer carboxylic acid (8:2 FTCA)	6<br>5
Fluorotelomer sulfonic acids	FTS	4:2 fluorotelomer sulfonic acid (4:2 FTS)<br>6:2 fluorotelomer sulfonic acid (6:2 FTS)	2<br>12
Perfluoroalkyl sulphonamides	FASA	Perfluorooctane sulphonamide (PFOSA/FOSA)<br>N-ethylperfluorooctane sulphonamide (N-EtFOSA)	20<br>11
Perfluoroalkane sulphonamido acetic acids	FASAA	N-methylperfluoro-1-octanesulfonamidoacetic acid (N-MeFOSAA)<br>N-ethylperfluoro-1-octanesulfonamidoacetic acid (N-EtFOSAA)	6<br>10
Perfluoroalkane sulphonamido ethanols	FASE	N-methylperfluorooctane sulphonamido ethanol (N-MeFOSE)<br>N-ethylperfluorooctane sulphonamido ethanol (N-EtFOSE)	4<br>4
Other PFAS		Polytetrafluoroethylene (PTFE)<br>Polyfluoroalkyl phosphate diesters (diPAPs)<br>4,8-dioxa 3H perfluorononanoic acid (ADONA)<br>Perfluoro-2-propoxypropanoic acid (HFPO-DA /Gen-X)	0<br>11<br>4<br>12

Table S2 - Estimated daily intakes calculated by previous studies using concentrations in foods, local consumption data and average body weight

Authors	Location	Plants studied	Soil concentrations (µg/kg, d.w.)	PFAS investigated/ reported	EDI from individual foods (ng/kg b.w./day)
[2]	China[a]	Tomato, cucumber	≤ 50	PFBA	0.27 – 2.62
				PFPeA	
				PFHxA	
				PFHpA	
				PFOA	0.01 – 0.06
				PFBS	0.05 – 0.40
				PFHxS	
				PFOS	
[3]	China[a]	Tomato, cucumber, eggplant, capsicum (pepper), Chinese cabbage	≤ 42	PFBA	4.0 – 207[b]
				PFPeA	
				PFHxA	
				PFHpA	
				PFOA	0.63 – 9.5[b]
				PFNA	
				PFDA	
				PFBS	0.42 - 34[b]
				PFHxS	
				PFOS	
[4]	Korea[a]	Apricot, white cabbage, Chinese cabbage, green onion, parsley, lettuce, rice, plum, raspberry, spinach, tomato	≤ 1.322	PFOA	<0.001 – 0.247<br>Sum 0.530[b]
				PFOS	<0.001 – 0.086<br>Sum 0.144[b]
[5]	China[a]	Lettuce, Chinese cabbage, chrysanthemum, cucumber	0.5 – 10 µg/L (hydroponic)		Approx. values[c]
				PFBA	4.6 – 96.2
				PFPeA	4.3 – 104
				PFHxA	1.4 – 26.8
				PFHpA	0.6 – 9
				PFOA	0.4 – 7
				PFNA	0.3 – 10.6
				PFDA	0.2 – 10.6
				PFUnDA	0.05 – 6.1
				PFDoDA	0 – 2.9
				HPFO-DA	0.3 – 7
				HPFO-TA	0 – 0.6
				PFBS	0 – 5
				PFHxS	0.2 – 2.7
				PFOS	0.15 – 5.6
				6:2 FTSA	1.4 – 2.9
				8:2 FTSA	0.3 – 10.6
				8:2 Cl-PFESA	0 – 0.3
[6]	Korea	Radish, onion, spring onion, beansprouts, spinach, kim chi, potato, sweet potato, cucumber, green pumpkin, cabbage	—[d]	PFBA	
				PFPeA	
				PFHxA	
				PFHpA	
				PFOA	0.17 – 18.43
				PFNA	
				PFDA	
				PFUnDA	
				PFDoDA	
				PFTrDA	
				PFTeDA	
				PFBS	
				PFHxS	
				PFOS	0.47 – 5.10
				PFDS	
					Sum PFAS

					4.23 – 86.97
7	Europe: Belgium, Czech Republic, Italy, Norway	Carrot, onion, tomato, courgette, cucumber, aubergine, peppers, cauliflower, cabbage, broccoli, lettuce variants, spinach, chicory, asparagus, celery, fennel, mushrooms, potato, peas, beans	—[d]	PFHxA	Approx. values[c]
				PFHpA	
				PFOA	0.027 – 0.185
				PFNA	
				PFDA	
				PFUnDA	
				PFDoDA	
				PFTrDA	
				PFTeDA	
				PFBA	
				PFHxS	
				PFOS	
				FOSA	
8 *Same concentration data as 7 above*	Europe: Belgium, Czech Republic, Italy, Norway	As above	—[d]	PFHxA	0.007 – 1.137
				PFHpA	
				PFOA	0.016 – 0.389
				PFNA	0.020 – 0.402
				PFDA	0.013 – 0.237
				PFUnDA	0.015 – 0.651
				PFDoDA	
				PFTrDA	
				PFTeDA	
				PFBA	
				PFHxS	0.001 – 0.329
				PFOS	0.080 – 1.113
				FOSA	
9	China[a]	White melon, white gourd, okra, cucumber, amaranth, Chinese little greens, tomato, gourd, leek, loofah, eggplant, pumpkin, spinach, peach, watermelon, grape, pear, muskmelon, pitaya	≤22.4	PFBA	4.2 – 26.3[b]
				PFPeA	
				PFHxA	
				PFHpA	
				PFOA	10.1 – 13.2
				PFNA	
				PFDA	
				PFUnDA	
				PFDoDA	
				PFTrDA	
				PFTeDA	
				PFHxA	
				PFDOA	
				PFBS	
				PFPeS	
				PFHxS	
				PFHpS	
				PFOS	
				PFNS	
				PFDS	
				PFDoDS	
					Total PFAS<br>15.9 - 39
10	China[a]	Wheat	PFOA ≤623[e]<br><br>Other PFAS ≤35.5	PFBA	10.73 – 1,219
				PFPeA	2.53 – 256.4
				PFHxA	1.09 – 141.5
				PFHpA	0.38 – 10.11
				PFOA	3.45 – 44.9
				PFNA	
				PFDA	
				PFUnDA	
				PFDoDA	
				PFBS	
				PFHxS	
				PFOS	
					Total PFAS<br>19.82 – 1,682
		Maize		PFBA	0.15 – 5.63
				PFPeA	0.06 – 0.48

				PFHxA	0.13 – 0.96
				PFHpA	0.0. – 0.16
				PFOA	0.06 – 0.15
				PFNA	
				PFDA	
				PFUnDA	
				PFDoDA	
				PFBS	
				PFHxS	
				PFOS	
					Total PFAS<br>0.54 – 7.53
11	China[a]	Wheat, corn, radish, carrot, Chinese cabbage, Chinese chives, pepper, Welsh onion, cauliflower, pumpkin, celery, soybean, lettuce	≤181	PFBA	4.1 – 2,743[b]
				PFPeA	0.94 – 419[b]
				PFHxA	0.75 – 94.7[b]
				PFHpA	0.76 – 42.9[b]
				PFOA	3.39 – 305[b]
				PFNA	
				PFDA	
				PFUnDA	
				PFDoDA	
				PFBS	
				PFHxS	
				PFOS	
					Total PFAS<br>11.5 – 3,544
12	China[a]	Cabbage (used as representative for all vegetable consumption)	≤82.6	PFBA	3.5 – 15.05
				PFPeA	1.33 – 6.48
				PFHxA	0.2 – 1
				PFHpA	0.07 – 0.3
				PFOA	0.16 – 0.25
				PFNA	
				PFDA	
				PFUnDA	
				PFDoDA	
				PFBS	0.05 – 0.38
				PFHxS	0.01
				PFOS	0.13 – 0.19
				HFPO-DA	0.28 – 0.99
				ADONA	
				6:2 FTS	0.08 – 0.17
				HFPO-TA	
				F-53B	0.77 – 1.32
					Total PFAS<br>6.59 – 26.16
13	China[a]	Cucumber, sponge gourd, tomato, eggplant, sweet pepper, balsam pear, zucchini, cabbage, spinach, rape, radish, carrot	≤2.09	PFBA	
				PFPeA	
				PFHxA	
				PFHpA	
				PFOA	0.308 – 0.543[b]
				PFNA	
				PFDA	
				PFUnDA	
				PFDoDA	
				PFBS	
				PFHxS	
				PFOS	
					Total PFAS<br>0.31 – 0.633[b]

[a] Crops contaminated by nearby industry or experimental design
[b] EDI range given is the sum of foods investigated
[c] Plant concentrations estimated from graph since exact values were not provided
[d] Soil concentrations not shown as produce was taken from market basket surveys
[e] Sites not at the fluorochemical plant location, or irrigated by the river directly downstream from it had PFOA concentrations ≤123.6

Table S3 – EDI for Australian adults based on food concentrations reported in literature

Food	EDI (ng/kg b.w. /day) [a]												Plant concentration source
	PFBA	PFPeA	PFHxA	PFHpA	PFOA	PFNA	PFDA	PFUnDA	PFDoDA	PFBS	PFHxS	PFOS	
Apple	0.04	n.d.	n.d.	n.d.	0.02	n.d.	n.d.					<0.01	[14]
Asparagus			0.09	n.d.	0.15	n.d.	n.d.	n.d.	n.d.	n.d.	n.d.	0.05	[7]
Banana	5.49	n.d.	n.d.	n.d.	n.d.	n.d.	n.d.					n.d.	[14]
Beans			0.17	0.07	0.23	n.d.	n.d.	n.d.	n.d.	n.d.	n.d.	0.07	[7]
Beetroot	n.d.	n.d.	n.d.	n.d.	<0.01	n.d.	n.d.					n.d.	[14]
Blackberry	<0.01	n.d.	n.d.	n.d.	<0.01	n.d.	n.d.	n.d.	n.d.	n.d.	n.d.	<0.01	[15]
Broccoli			n.d.	n.d.	0.59	0.35	n.d.	n.d.	n.d.	n.d.	n.d.	<0.01	[7]
Cabbage [b]	0.01 – 6.85	<0.01 – 1.72	<0.01 – 0.37	<0.01 – 0.37	<0.01 – 4.01	<0.01	<0.01	<0.01	<0.01	n.d.	n.d.	<0.01 – 0.57	[4, 7, 11, 13, 14]
Capsicum (pepper)	0.01 – 6.00	<0.01 – 2.64	<0.01 – 0.63	<0.01 – 0.11	<0.01 – 3.23	<0.01	<0.01	<0.01	<0.01	<0.01	<0.01	<0.01	[7, 13]
Carrot	0.04 – 9.07	<0.01 – 1.13	<0.01 – 1.67	<0.01 – 0.21	<0.01 – 8.99	<0.01	<0.01	n.d.	<0.01	<0.01	<0.01	<0.01 – 1.01	[7, 11, 13, 14]
Cauliflower	0.01 – 1.63	<0.01 – 0.66	<0.01 – 0.28	<0.01 – 0.16	<0.01 – 0.72	<0.01	<0.01	n.d.	<0.01	n.d.	<0.01	<0.01	[7, 11]
Celery	<0.01 – 1.58	0.01 – 0.54	<0.01 – 0.50	<0.01 – 0.08	<0.01 – 1.18	<0.01 – 0.05	<0.01 – 0.11	n.d.	n.d.	<0.01 – 0.39	<0.01 – 0.01	<0.01 – 0.25	[7, 11, 16]
Cherry	n.d.	n.d.	n.d.	n.d.	<0.01	n.d.	n.d.					n.d.	[14]
Corn (maize)	<0.01 – 0.44	<0.01 – 0.09	<0.01 – 0.15	<0.01	<0.01 – 0.09	<0.01	<0.01	<0.01	<0.01	<0.01	<0.01	<0.01	[10, 11, 17, 18]
Cucumber	<0.01 – 0.24	<0.01 – 0.02	<0.01	<0.01	<0.01 – 2.19	<0.01	<0.01 – 0.25	<0.01	<0.01	<0.01 – 0.06	<0.01	<0.01	[2, 7, 13]
Eggplant	<0.01	<0.01	<0.01	n.d.	<0.01	<0.01	<0.01	<0.01	<0.01	n.d.	n.d.	n.d.	[13]
Fennel			n.d.	0.16	0.05	0.02	n.d.	n.d.	n.d.	n.d.	n.d.	n.d.	[7]
Lemon	<0.01	n.d.	n.d.	n.d.	<0.01	n.d.	n.d.					n.d.	[14]
Lettuce	0.06 – 33.2	<0.01 – 3.95	<0.01 – 3.07	<0.01 – 1.02	<0.01 – 14.6	<0.01 – 0.81	<0.01 – 0.68	<0.01 – 0.18	<0.01 – 0.19	<0.01 – 2.88	<0.01 – 0.15	<0.01 – 1.43	[4, 7, 11, 19, 20]

Mushroom			0.49	n.d.	n.d.	0.34	n.d.	n.d.	n.d.	n.d.	n.d.	n.d.	[7]
Onion			2.68	n.d.	<0.01 – 6.85	1.19	n.d.	n.d.	n.d.	n.d.	n.d.	<0.01 – 6.95	[4, 7]
Orange	0.01	n.d.	n.d.	n.d.	0.01	n.d.	n.d.					n.d.	[14]
Pea	<0.01 – 0.19	<0.01 – 0.06	<0.01 – 0.02	<0.01	<0.01	<0.01	n.d.			<0.01 – 0.02	<0.01	<0.01	[16]
Plum					<0.01							n.d.	[4]
Potato			10.8	n.d.	<0.01 – 6.54	2.10	n.d.	n.d.	n.d.	n.d.	n.d.	n.d.	[7, 14]
Pumpkin	7.79	0.78	0.14	0.06	0.18	<0.01	n.d.	<0.01	n.d.	n.d.	n.d.	<0.01	[11]
Rice	n.d.	n.d.	n.d.	<0.01 – 0.05	<0.01 – 0.68	<0.01 – 0.08	<0.01 – 0.04	<0.01 – 0.04	<0.01 – 0.02	n.d.	n.d.	<0.01 – 0.02	[4, 21]
Spinach	<0.01	<0.01	<0.01 – 0.15	<0.01 – 0.10	<0.01 – 0.57	<0.01	<0.01	<0.01	<0.01	<0.01	<0.01	<0.01 – 0.09	[7, 13, 22]
Tomato	<0.01 – 1.69	<0.01 – 4.11	<0.01 – 3.38	<0.01 – 0.69	<0.01 – 4.33	<0.01	<0.01	<0.01	<0.01	<0.01 – 0.38	<0.01	<0.01	[2, 7, 13, 14, 19, 22]
Wheat	0.92 – 806	<0.35 – 111	<0.17 – 21.7	<0.24 – 7.85	0.06 – 45.9	<0.12 – 7.19	<0.17 – 2.24	<0.21 – 0.59	<0.14 – 0.40	<0.05 – 0.21	<0.05 – 10.3	<0.01 – 5.97	[10, 11, 18, 23]
Zucchini (squash)	<0.01	<0.01	<0.01	n.d.	<0.01	<0.01	n.d.	<0.01	<0.01	n.d.	n.d.	n.d.	[13]

[a] Low EDIs are given as <0.01 rather than actual values, as they were regarded as negligible.

[b] Includes white cabbage and Chinese cabbage, regular cabbage consumption was used for EDI calculations

n.d. – tested for but not detected in any study, this was left blank if compound was not tested for by any study.

Soil PFAS concentrations were significantly elevated for some locations impacted by a fluorochemical plant in [10]. Only food plant concentrations from the locations that were upstream of the fluorochemical plant were used for EDI calculations.

Table S4 – Per capita supply of food plants to Australian residents 2020-21 [24] and mean moisture content used to convert to dry weight consumption [25]

Food	Supply per capita (g, fresh weight)		Mean moisture content (%)	Supply per capita, daily (g, dry weight)
	Annual	Daily		
Apple	7800	21.4	85.6	3.1
Asparagus	314	0.86	93.2	0.06
Banana	16000	43.8	74.9	11.0
Bean	1300	3.56	90.3	0.3
Beetroot	203	0.56	87.6	0.07
Blackberry	99	1.08	27.1	0.04
Broccoli	2800	7.67	90.7	0.7
Cabbage	1900	5.21	91.0	0.5
Capsicum (Pepper)	3000	8.22	93.9	0.5
Carrot	8000	21.9	88.3	2.6
Cauliflower	3000	8.22	91.9	0.7
Celery	2300	6.30	95.4	0.3
Cherry	565	1.55	82.3	1.55
Corn (maize)	1400	3.84	76.0	0.9
Cucumber	3300	9.04	96.7	0.3
Eggplant	333	0.91	92.4	0.07
Fennel	52	0.14	N/A[a]	N/A[a]
Lemon	1540	4.22	89.0	0.5
Lettuce	5400	14.8	95.6	0.6
Mushroom	2800	7.67	88.8	0.9
Onion	8000	21.9	89.1	2.4
Orange	5100	14.0	86.8	1.9
Pea	322	0.88	88.9	0.1
Plum	635	1.74	87.2	0.22
Potato	18000	49.3	81.6	9.1
Pumpkin	4200	11.5	91.6	0.97
Rice	13500[b]	37.0	1.6	36.4
Spinach	239	0.65	87.9	0.08
Tomato	9300	25.5	94.0	1.5
Wheat	75400[c]	206.6	9.6	186.8

[a] Moisture data not given

[b] Per capita consumption from [26]

[c] Per capita consumption from [27]

No allowance made for waste, processing into different forms, or variability of consumption by age or sex since that data was unavailable. Only foods with supply data and related to plant uptake studies conducted in non-spiked soils from locations not deemed highly contaminated are listed.

Table S5 – Per capita consumption of different food types by Australians [28], Americans {Statista 2023} and Chinese separated by region {Song 2017}

Food	Daily per capita consumption (g)			
	Australia	United States	Northern China	Southern China
Meat: Fish / Seafood	11.7	19.9	18.1	46.1
Red meat	41.1	139	43.9	90.2
Eggs	17.1	43.6	28.2	16.9
Dairy: Milk	263	62.2	32.3	32.4
Cheese	23.6	51.8	0.3[a]	0.3[a]
Yoghurt	21.4	17.3	18.3[a]	18.3[a]
Food plants: Vegetables	179	173	224	291
Fruit	185	144	42.3	32.4
Rice	37.0	39.7	123	328
Wheat	210	218	261	63.1

[a] {Statista 2023} Dairy consumption in 2022 was approximately the same as 2017

Table S6 – Per capita consumption of food plants by residents of the United States 2021-22 [29]

Food	Daily consumption per capita (g, fresh weight)
Apple	19.6
Asparagus	2.09
Banana	33.1
Bean	1.50
Blackberry	0.32
Broccoli	6.51
Cabbage	7.68
Capsicum	13.7
Carrot	10.4
Cauliflower	2.64
Celery	5.16
Cherry	1.23
Cucumber	10.1
Eggplant	1.25
Lemon	5.88
Lettuce	28.7
Maize (corn)	5.11
Mushroom	4.61
Onion	23.8
Orange	9.55
Potato	121.7
Pumpkin	6.98
Rice	39.7[a]
Spinach	2.67
Squash	6.54
Tomato	23.7
Wheat	217.8[b]

[a] {Statista 2021}, [b] {Statista 2021}

Table S7 – EDI for United States adults based on food concentrations reported in literature

Food	EDI (ng/kg b.w. /day) [a]												Plant concentration source
	PFBA	PFPeA	PFHxA	PFHpA	PFOA	PFNA	PFDA	PFUnDA	PFDoDA	PFBS	PFHxS	PFOS	
Apple	0.04	n.d.	n.d.	n.d.	0.02	n.d.	n.d.					<0.01	[14]
Asparagus			0.20	n.d.	0.34	n.d.	n.d.	n.d.	n.d.	n.d.	n.d.	0.11	[7]
Banana	3.91	n.d.	n.d.	n.d.	n.d.	n.d.	n.d.					n.d.	[14]
Beans			0.07	0.03	0.09	n.d.	n.d.	n.d.	n.d.	n.d.	n.d.	0.03	[7]
Blackberry	<0.01	n.d.	n.d.	n.d.	<0.01	n.d.	n.d.	n.d.	n.d.	n.d.	n.d.	<0.01	[15]
Broccoli			n.d.	n.d.	0.47	0.29	n.d.	n.d.	n.d.	n.d.	n.d.	n.d.	[7]
Cabbage [b]	0.02 – 9.53	<0.01 – 2.39	<0.01 – 0.52	<0.01 – 0.51	<0.01 – 5.58	<0.01	<0.01	<0.01	<0.01	<0.01	<0.01	<0.01 – 0.79	[4, 7, 11, 13, 14]
Capsicum (pepper)	0.01 – 9.46	<0.01 – 4.16	<0.01 – 1.00	<0.01 – 0.18	<0.01 – 5.10	<0.01	<0.01	<0.01	<0.01	<0.01	<0.0.1	<0.01	[7, 13]
Carrot	0.02 – 4.07	<0.01 – 0.51	<0.01 – 0.75	<0.01 – 0.09	<0.01 – 4.03	<0.01	<0.01	n.d.	<0.01	<0.01	<0.01	<0.01 – 0.45	[7, 11, 13, 14]
Cauliflower	<0.01 – 0.49	<0.01 – 0.20	<0.01 – 0.08	<0.01 – 0.05	<0.01 – 0.22	<0.01	<0.01	n.d.	<0.01	n.d.	<0.01	<0.01	[7, 11]
Celery	<0.01 – 1.22	<0.01 – 0.42	<0.01 – 0.38	<0.01 – 0.06	<0.01 – 0.91	<0.01 – 0.04	<0.01 – 0.08	n.d.	n.d.	<0.01 – 0.30	<0.01	<0.01 – 0.19	[7, 11, 16]
Cherry	n.d.	n.d.	n.d.	n.d.	<0.01	n.d.	n.d.					n.d.	[14]
Corn (maize)	<0.01 – 0.55	<0.01 – 0.11	<0.01 – 0.19	<0.01	<0.01 – 0.12	<0.01	<0.01	<0.01	<0.01	<0.01	<0.01 – 0.01	<0.01 – 0.01	[10, 11, 17, 18]
Cucumber	<0.01 – 0.25	<0.01 – 0.02	<0.01	<0.01	<0.01 – 2.31	<0.01	0.26	<0.01	<0.01 – 0.26	<0.01 – 0.06	<0.01	<0.01	[2, 7, 13]
Eggplant	<0.01	<0.01	<0.01	n.d.	<0.01	<0.01	<0.01	<0.01	<0.01	n.d.	n.d.	n.d.	[13]
Lemon	<0.01	n.d.	n.d.	n.d.	0.01	n.d.	n.d.					n.d.	[14]
Lettuce	0.06 – 39.53	<0.01 – 4.70	<0.01 – 3.65	<0.01 – 1.22	<0.01 – 17.35	<0.01 – 0.96	<0.01 – 0.81	<0.01 – 0.22	<0.01 – 0.23	<0.01 – 3.43	<0.01 – 0.17	<0.01 – 1.70	[4, 7, 11, 19, 20]
Mushroom			0.28	n.d.	n.d.	0.19	n.d.	n.d.	n.d.	n.d.	n.d.	n.d.	[7]
Onion			2.75	n.d.	7.02	1.22	n.d.	n.d.	n.d.	n.d.	n.d.	7.12	[7]
Orange	<0.01	n.d.	n.d.	n.d.	<0.01	n.d.	n.d.					n.d.	[14]
Potato			24.9	n.d.	0.02 – 15.1	4.84	n.d.	n.d.	n.d.	n.d.	n.d.	n.d.	[7, 14]
Pumpkin	4.46	0.45	0.08	0.04	0.11	<0.01	n.d.	<0.01	n.d.	n.d.	n.d.	<0.01	[11]

Rice	n.d.	n.d.	n.d.	<0.01 – 0.05	<0.01 – 0.68	<0.01 – 0.08	<0.01 – 0.05	<0.01 – 0.04	<0.01 – 0.02	n.d.	n.d.	<0.01 – 0.02	4, 21
Spinach	<0.01 – 0.02	<0.01	<0.01 – 0.57	<0.01 – 0.37	<0.01 – 2.18	<0.01	<0.01	<0.01	<0.01	<0.01	<0.01	<0.01 – 0.34	7, 13, 22
Tomato	<0.01 – 1.48	<0.01 – 3.60	<0.01 – 2.95	<0.01 – 0.13	<0.01 – 3.79	<0.01	<0.01	<0.01	<0.01	<0.01 – 0.33	<0.01	<0.01	2, 7, 13, 14, 19, 22
Wheat	0.91 – 801	<0.35 – 110	<0.16 – 21.6	<0.23 – 7.81	0.06 – 45.7	<0.12 – 7.15	<0.16 – 2.23	<0.21 – 0.59	<0.14 – 0.40	<0.05 – 0.21	<0.05 – 10.2	<0.01 – 5.93	10, 11, 18, 23
Zucchini (squash)	<0.01	<0.01	<0.01	n.d.	<0.01	<0.01	n.d.	<0.01	<0.01	n.d.	n.d.	n.d.	13

[a] Low EDIs are given as <0.01 rather than actual values, as they were regarded as negligible.

[b] Includes white cabbage and Chinese cabbage, regular cabbage consumption was used for EDI calculations

n.d. – tested for but not detected in any study, no value given if compound was not tested for by any study.

The soil PFAS concentrations were significantly elevated for some locations impacted by a fluorochemical plant in [10], only plant concentrations from the locations that weren’t downstream of the plant were used for EDI calculation

Table S8 – EDI of PFAS for United States adults from all sources where data is available

Source	EDI (ng/kg b.w./day)												PFAS concentration reference
	PFBA	PFPeA	PFHxA	PFHpA	PFOA	PFNA	PFDA	PFUnDA	PFDoDA	PFBS	PFHxS	PFOS	
Food plants	9.48 – 872	0.47 – 127	28.3 – 57.6	0.07 – 10.5	8.21 – 111	6.82 – 14.8	0.21 – 3.44	<0.01 – 0.85	<0.01 – 0.66	<0.01 – 4.34	<0.01 – 10.5	0.15 – 16.7	This review
Fish / seafood	<0.01 – 0.28	<0.01 – 0.61	<0.01 – 0.24	<0.01 – 0.10	<0.01 – 4.77	<0.01 – 0.57	<0.01 – 0.28	<0.01 – 1.01	<0.01 – 0.24	<0.01 – 0.02	<0.01 – 0.17	<0.01 – 4.52	[30]
Red meat	<0.01	<0.01	<0.01	<0.01 – 0.10	<0.01 – 0.17	<0.01	<0.01	<0.01 – 0.11	<0.01	<0.01	<0.01	<0.01 – 0.14	[31]
Eggs			<0.13	<0.26	<0.13 – 1.40	<0.13 – 1.04	<0.26 – 4.15	<0.26 – 2.34		<0.26	<0.13 – 2.70	<0.13 – 12.9	[32]
Dairy	<0.01 – 1.36	0.02 – 0.05	0.02 – 0.06	0.02 – 0.22	<0.01 – 5.31	<0.01 – 1.76	<0.01 – 2.36	<0.01 – 1.45	<0.01 – 2.05	<0.01 – 0.03	0.03 – 0.09	0.02 – 4.66	[31, 33]
Water	0.02 – 3.80	0.02 – 0.37	<0.01 – 0.33	<0.01 – 0.13	0.01 – 0.40	<0.01 – 0.15	0.03 – 0.03			<0.01 – 0.50	<0.01 – 0.53	<0.01 – 0.45	[34]
Dust ingestion	<0.01 – 0.04	<0.01 – 0.01	<0.01 – 0.03	<0.01 – 0.05	<0.01 – 0.11	<0.01 – 0.04	<0.01 – 0.07	<0.01 – 0.04	<0.01 – 0.03	<0.01 – 0.02	<0.01 – 0.29	<0.01 – 0.48	[35]
Inhalation			<0.01 – 0.10	<0.01 – 0.03	0.01 - 1.25	<0.01 – 1.05	<0.01 – 0.47	<0.01 – 0.04	<0.01 – 0.13			<0.01	[36, 37]
Dermal			<0.01	<0.01	<0.01 – 0.02	<0.01					<0.01 – 0.01	<0.01	[37]
Total	9.51 – 877	0.51 – 128	28.3 – 58.4	0.10 – 11.2	8.23 – 125	6.83 – 19.4	0.25 – 10.8	<0.01 – 5.84	<0.01 – 3.10	<0.01 – 4.90	0.03 – 14.2	0.16 – 39.8	

Table S9 – EDI for PFAS from food groups using average concentrations and consumption for China overall {Statista 2023}, or divided into North and South as reported by [38] along with other sources. Blank spaces indicate no testing for those compounds in the studies referenced.

Source	EDI (ng/kg b.w./day)												PFAS concentration reference
	PFBA	PFPeA	PFHxA	PFHpA	PFOA	PFNA	PFDA	PFUnDA	PFDoDA	PFBS	PFHxS	PFOS	
**Food plants:** Northern	2.04 – 95.2	0.51 – 27.8	0.77 – 9.91	0.19 – 1.23	1.13 – 8.80	0.15 – 0.88	0.11 – 0.48	0.13 – 0.43	0.06 – 0.24	0.03 – 0.10	0.06 – 0.17	0.11 – 0.68	This review
Southern	1.09 – 29.8	0.24 – 8.30	0.60 – 4.14	0.10 – 1.07	1.17 – 13.2	0.08 – 1.28	0.05 – 0.75	0.04 – 0.54	0.02 – 0.31	0.02 – 0.09	0.02 – 0.05	0.09 – 0.54	
**Fish / seafood:** Northern	<0.01 – 0.35	<0.01 – 0.75	<0.01 – 0.29	<0.01 – 0.12	<0.01 – 5.84	<0.01 – 0.69	<0.01 – 0.34	<0.01 – 1.24	<0.01 – 0.29	<0.01 – 0.02	<0.01 – 0.21	<0.01 – 5.54	[30]
Southern	<0.01 – 0.90	<0.01 – 1.94	<0.01 – 0.75	<0.01 – 0.31	<0.01 – 15.1	<0.01 – 1.80	<0.01 – 0.88	<0.01 – 3.21	<0.01 – 0.76	<0.01 – 0.05	<0.01 – 0.54	<0.01 – 14.3	
**Red meat:** Northern	<0.01	<0.01	<0.01	<0.01 – 0.04	<0.01 – 0.07	<0.01	<0.01	<0.01 – 0.05	<0.01	<0.01	<0.01	<0.01 – 0.06	[31]
Southern				<0.01 – 0.10	<0.01 – 0.16			<0.01 – 0.10				<0.01 – 0.13	
**Eggs:** Northern			<0.14	<0.29	<0.14 – 1.22	<0.14 – 0.90	<0.29 – 3.62	<0.29 – 2.03		<0.29	<0.14 – 2.35	<0.14 – 11.2	[32]
Southern					<0.14 – 0.80	<0.14 – 0.59	<0.29 – 2.36	<0.29 – 1.33			<0.14 – 1.53	<0.14 – 7.32	
**Dairy:** Northern	<0.01 – 1.26	0.02 – 0.11	0.02 – 0.04	0.02 – 0.16	<0.01 – 3.53	<0.01 – 1.22	<0.01 – 1.62	<0.01 – 1.01	<0.01 – 1.43	<0.01 – 0.02	<0.01 – 0.04	<0.01 – 3.22	[31, 33]
Southern	<0.01 – 1.15	0.02 – 0.08	0.02 – 0.04	0.02 – 0.12	<0.01 – 2.30	<0.01 – 0.80	<0.01 – 1.05	<0.01 – 0.65	<0.01 – 1.56	<0.01 – 0.01	<0.01 – 0.03	<0.01 – 2.08	
**Water:** Northern	<0.01 – 0.88	<0.01 – 0.04	<0.01 – 0.13	<0.01 – 0.05	<0.01 – 0.56	<0.01 – 0.19	<0.01 – 0.02	<0.01 – 0.01	<0.01 – 0.02	<0.01 – 0.10	<0.01 – 0.58	<0.01 – 0.32	[39]
Southern	<0.01 – 3.98	<0.01 – 0.15	<0.01 – 2.50	<0.01 – 0.24	<0.01 – 16.4	<0.01 – 0.69	<0.01 – 0.12	<0.01 – 0.01	<0.01 – 0.02	<0.01 – 0.31	<0.01 – 1.06	<0.01 – 6.51	
**Dust ingestion**	<0.01 – 0.04	<0.01 – 0.01	<0.01 – 0.03	<0.01 – 0.05	<0.01 – 0.11	<0.01 – 0.04	<0.01 – 0.07	<0.01 – 0.04	<0.01 – 0.03	<0.01 – 0.02	<0.01 – 0.29	<0.01 – 0.48	[35]
**Inhalation**			<0.01 – 0.10	<0.01 – 0.03	0.01 - 1.25	<0.01 – 1.05	<0.01 – 0.47	<0.01 – 0.04	<0.01 – 0.13			<0.01	[36, 37]
**Dermal**			<0.01	<0.01	<0.01 – 0.02	<0.01					<0.01 – 0.01	<0.01	[37]
**Total:** Northern	2.04 – 97.8	0.53 – 28.7	0.79 – 10.5	0.21 – 1.63	1.15 – 21.6	0.15 – 4.95	0.11 – 6.56	0.13 – 4.81	0.06 – 2.11	0.03 – 0.26	0.06 – 3.36	0.11 – 21.0	
Southern	1.09 – 35.9	0.26 – 10.5	0.62 – 7.53	0.12 – 1.86	1.19 – 49.3	0.08 – 6.20	0.05 – 5.63	0.04 – 5.87	0.02 – 2.78	0.02 – 0.47	0.02 – 3.23	0.09 – 31.0	

Table S10 – EDI for PFAS from food plants using grouped average concentrations and consumption for Australia for comparison to China's overall EDI in Table S9

EDI (ng/kg b.w./day)											
PFBA	PFPeA	PFHxA	PFHpA	PFOA	PFNA	PFDA	PFUnDA	PFDoDA	PFBS	PFHxS	PFOS
1.37 – 60.6	0.32 – 17.7	0.49 – 6.29	0.12 – 0.69	0.87 – 4.38	0.09 – 0.40	0.07 – 0.21	0.08 – 0.20	0.04 – 0.11	0.02 – 0.07	0.04 – 10	0.09 – 42

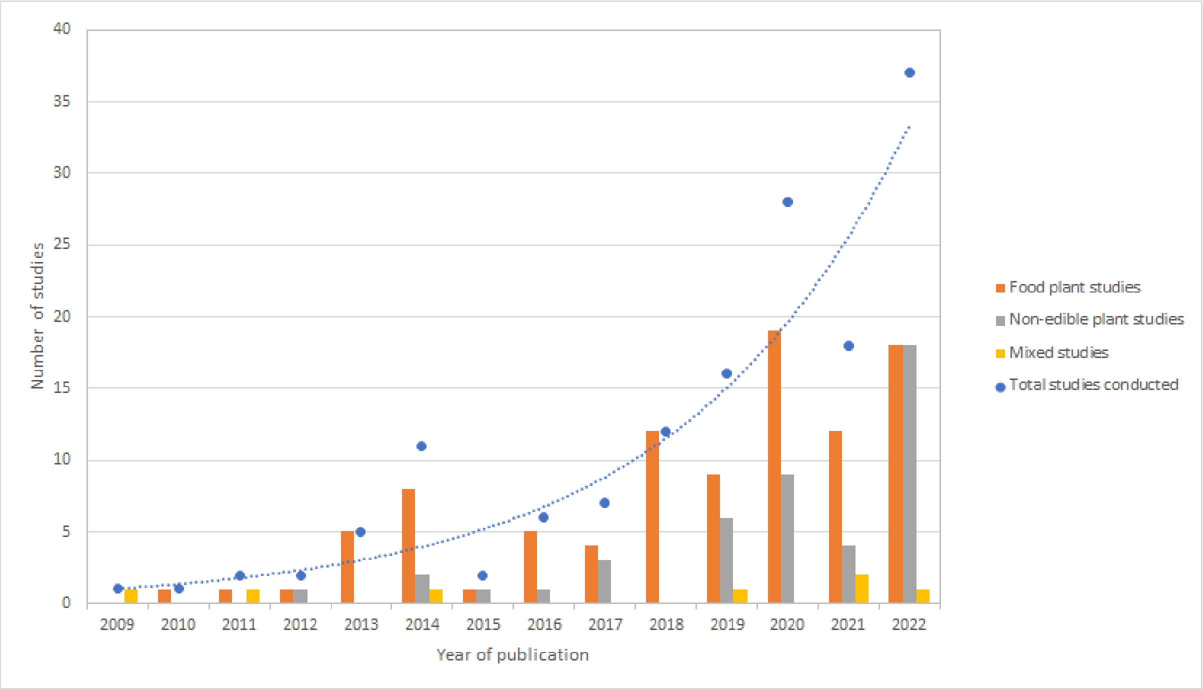

Figure S1 – Studies found to report plant uptake concentrations of PFAS over time as of 15 February 2023

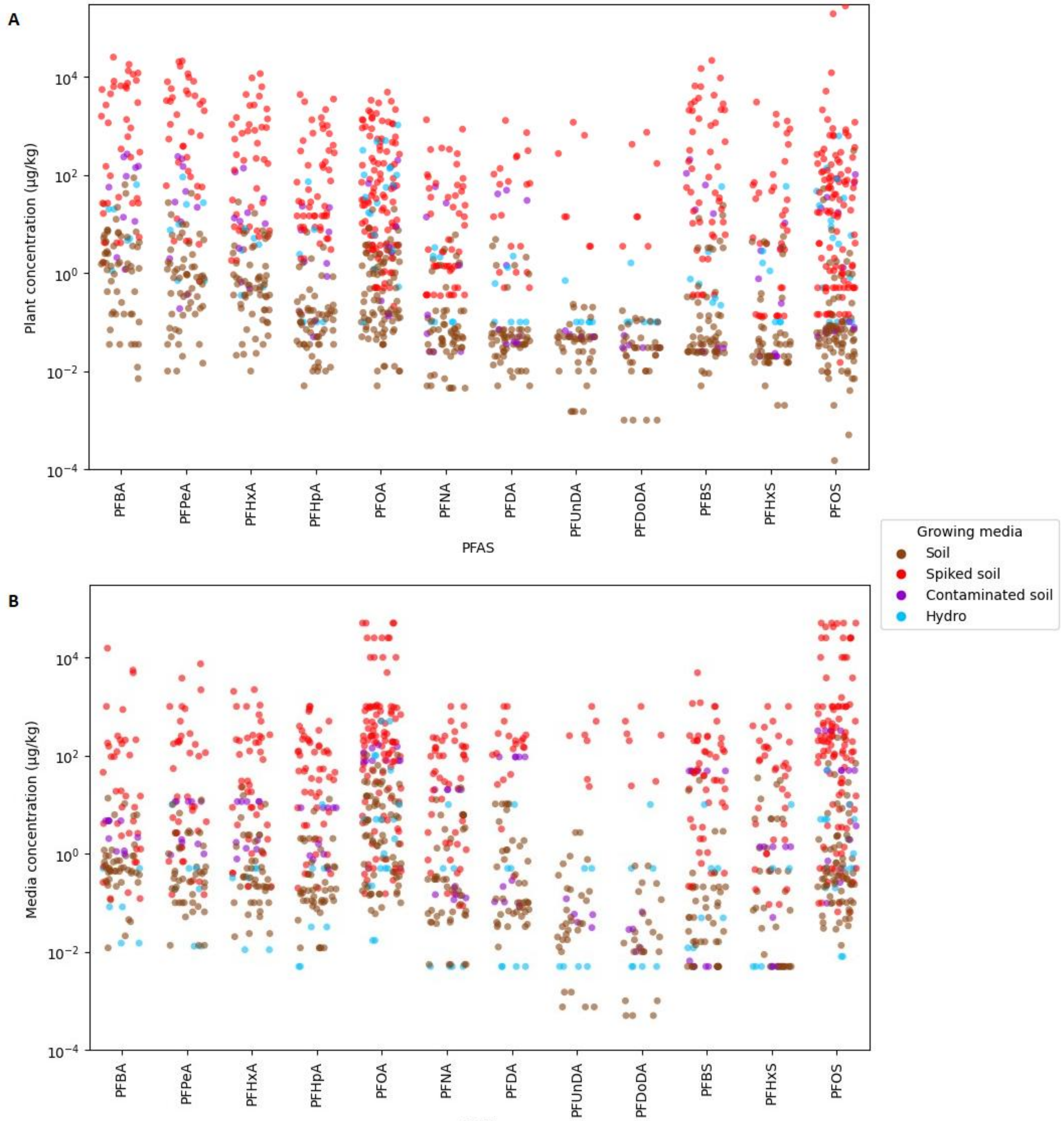

Figure S2 – Strip plot with jitter showing reported PFAS concentrations in edible portions of food plants (A) and PFAS concentrations of the media they were grown in (B). Dot colour indicates the type of growing media, brown dots show soils that had not been spiked or impacted by PFAS contamination from industry. Average concentrations were used where multiple trials of the same conditions were reported, and ½ LOD was used where compounds were present in the growing media but not detected in plants.

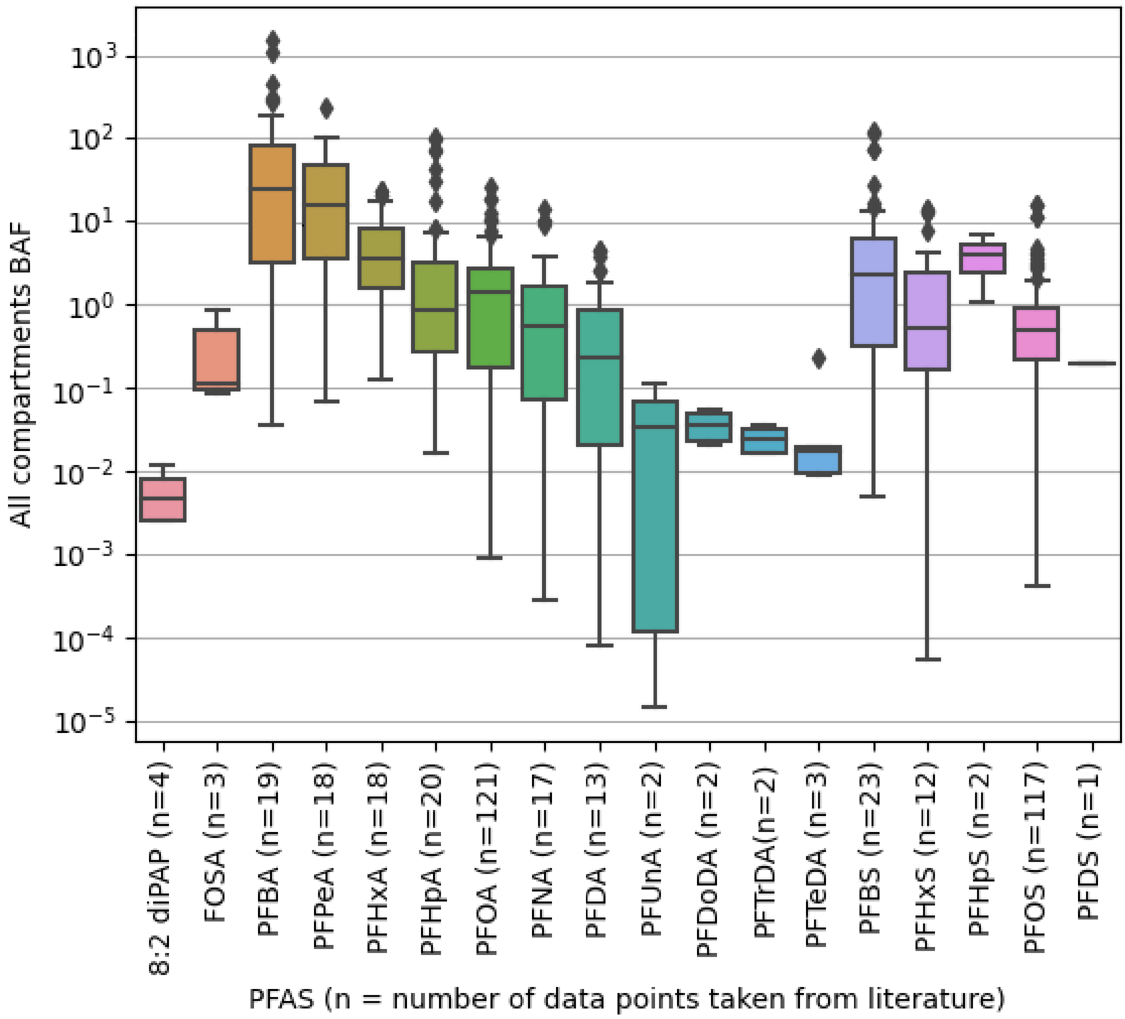

Figure S3 – Combined BAF in reviewed literature for all edible compartments shown in Figure 1

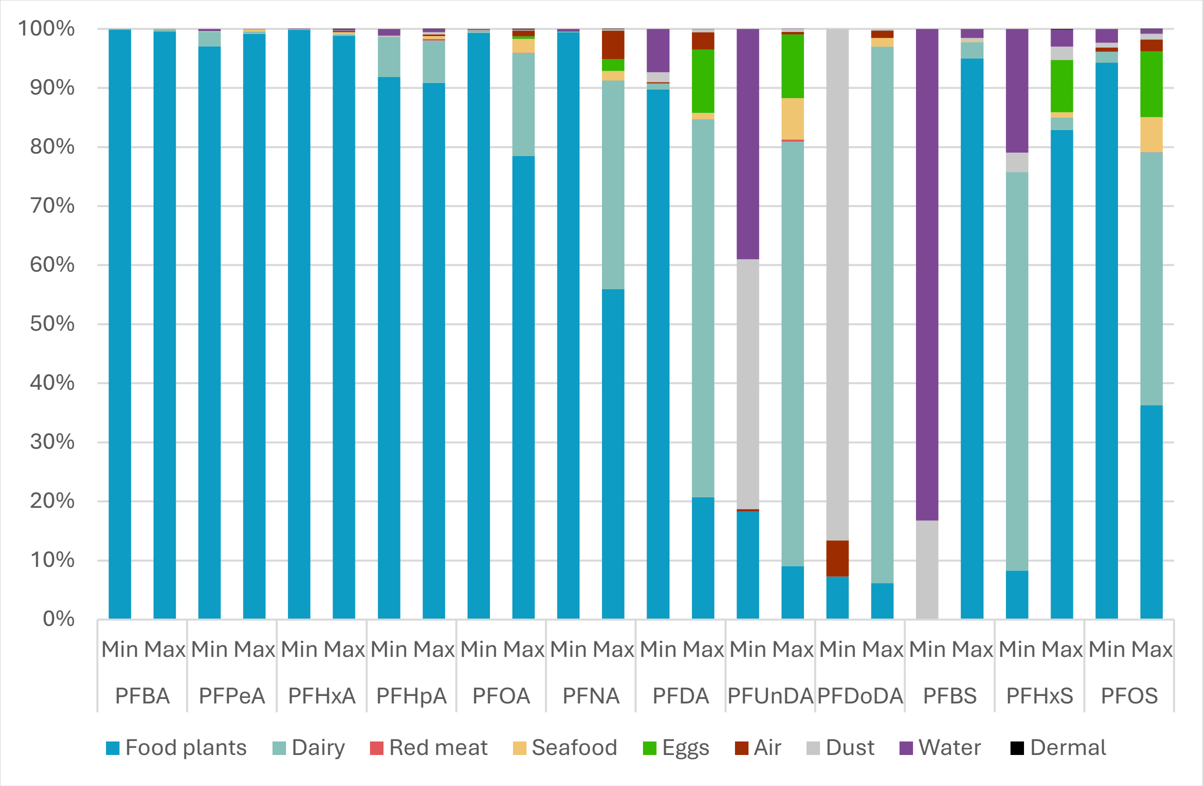

Figure S4 – Percentage contribution of different PFAS sources to total EDI maximum and minimum

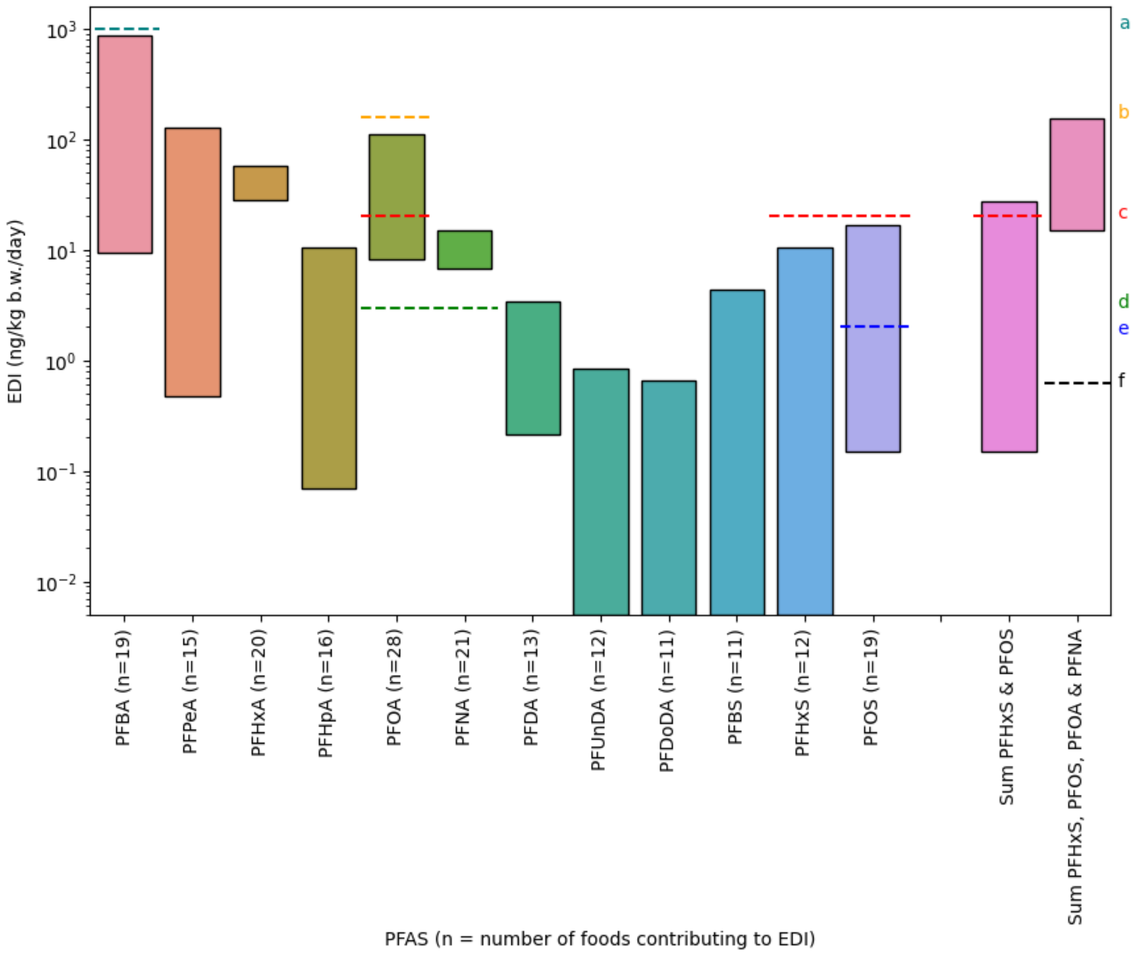

Figure S5 – Range of total EDIs for United States adults from food plants based on minimum and maximum reported plant concentrations in uptake studies with <100 µg/kg PFAS concentrations. PFDoDA, PFBS and PFHxS were below LOD in at least one instance for each food plant reported.

[a] Reference dose for PFBA [40] (1000 ng/kg b.w./day)

[b] Tolerable Daily Intake (TDI) for PFOA [41] (160 ng/kg b.w./day)

[c] Reference dose for PFOA and PFOS, [42] TDI of the sum of PFOS and PFHxS [41] and Minimum Risk Level (MRL) for PFHxS [43] (20 ng/kg b.w./day)

[d] MRL for PFOA and PFNA [43] (3 ng/kg b.w./day)

[e] MRL for PFOS [43] (2 ng/kg b.w./day)

[f] TDI of the sum of PFOA, PFNA, PFHxS and PFOS [29] (0.63 ng/kg b.w./day)

References

(1) Dasu, K.; Xia, X.; Siriwardena, D.; Klupinski, T. P.; Seay, B. Concentration profiles of per- and polyfluoroalkyl substances in major sources to the environment. *Journal of Environmental Management* **2022**, *301*, 113879. DOI: 10.1016/j.jenvman.2021.113879.
(2) Bao, J.; Li, C. L.; Liu, Y.; Wang, X.; Yu, W. J.; Liu, Z. Q.; Shao, L. X.; Jin, Y. H. Bioaccumulation of perfluoroalkyl substances in greenhouse vegetables with long-term groundwater irrigation near fluorochemical plants in Fuxin, China. *Environmental Research* **2020**, *188*, 109751. DOI: 10.1016/j.envres.2020.109751 From NLM Medline.
(3) Bao, J.; Yu, W. J.; Liu, Y.; Wang, X.; Jin, Y. H.; Dong, G. H. Perfluoroalkyl substances in groundwater and home-produced vegetables and eggs around a fluorochemical industrial park in China. *Ecotoxicology & Environmental Safety* **2019**, *171*, 199-205. DOI: 10.1016/j.ecoenv.2018.12.086.
(4) Choi, G. H.; Lee, D. Y.; Bruce-Vanderpuije, P.; Song, A. R.; Lee, H. S.; Park, S. W.; Lee, J. H.; Megson, D.; Kim, J. H. Environmental and dietary exposure of perfluorooctanoic acid and perfluorooctanesulfonic acid in the Nakdong River, Korea. *Environmental Geochemistry Health* **2021**, *43* (1), 347-360. DOI: 10.1007/s10653-020-00721-0.
(5) Gu, Q.; Wen, Y.; Wu, H.; Cui, X. Uptake and translocation of both legacy and emerging per- and polyfluorinated alkyl substances in hydroponic vegetables. *Sci Total Environ* **2023**, *862*, 160684. DOI: 10.1016/j.scitotenv.2022.160684.
(6) Heo, J.-J.; Lee, J.-W.; Kim, S.-K.; Oh, J.-E. Foodstuff analyses show that seafood and water are major perfluoroalkyl acids (PFAAs) sources to humans in Korea. *Journal of hazardous materials* **2014**, *279*, 402-409.
(7) Herzke, D.; Huber, S.; Bervoets, L.; D'Hollander, W.; Hajslova, J.; Pulkrabova, J.; Brambilla, G.; De Filippis, S. P.; Klenow, S.; Heinemeyer, G.; et al. Perfluorinated alkylated substances in vegetables collected in four European countries; occurrence and human exposure estimations. *Environ Sci Pollut Res Int* **2013**, *20* (11), 7930-7939. DOI: 10.1007/s11356-013-1777-8.
(8) Klenow, S.; Heinemeyer, G.; Brambilla, G.; Dellatte, E.; Herzke, D.; de Voogt, P. Dietary exposure to selected perfluoroalkyl acids (PFAAs) in four European regions. *Food Addit Contam Part A Chem Anal Control Expo Risk Assess* **2013**, *30* (12), 2141-2151. DOI: 10.1080/19440049.2013.849006.
(9) Li, P.; Oyang, X.; Zhao, Y.; Tu, T.; Tian, X.; Li, L.; Zhao, Y.; Li, J.; Xiao, Z. Occurrence of perfluorinated compounds in agricultural environment, vegetables, and fruits in regions influenced by a fluorine-chemical industrial park in China. *Chemosphere* **2019**, *225*, 659-667. DOI: 10.1016/j.chemosphere.2019.03.045.
(10) Liu, Z.; Lu, Y.; Shi, Y.; Wang, P.; Jones, K.; Sweetman, A. J.; Johnson, A. C.; Zhang, M.; Zhou, Y.; Lu, X.; et al. Crop bioaccumulation and human exposure of perfluoroalkyl acids through multi-media transport from a mega fluorochemical industrial park, China. *Environ Int* **2017**, *106*, 37-47. DOI: 10.1016/j.envint.2017.05.014.
(11) Liu, Z.; Lu, Y.; Song, X.; Jones, K.; Sweetman, A. J.; Johnson, A. C.; Zhang, M.; Lu, X.; Su, C. Multiple crop bioaccumulation and human exposure of perfluoroalkyl substances around a mega fluorochemical industrial park, China: Implication for planting optimization and food safety. *Environment International* **2019**, *127*, 671-684. DOI: 10.1016/j.envint.2019.04.008 From NLM Medline.
(12) Xu, C.; Song, X.; Liu, Z.; Ding, X.; Chen, H.; Ding, D. Occurrence, source apportionment, plant bioaccumulation and human exposure of legacy and emerging per-and polyfluoroalkyl substances in soil and plant leaves near a landfill in China. *Science of the Total Environment* **2021**, *776*, 145731.
(13) Zhang, M.; Wang, P.; Lu, Y.; Lu, X.; Zhang, A.; Liu, Z.; Zhang, Y.; Khan, K.; Sarvajayakesavalu, S. Bioaccumulation and human exposure of perfluoroalkyl acids (PFAAs) in vegetables from the

largest vegetable production base of China. *Environment International* **2020**, *135*, 105347. DOI: 10.1016/j.envint.2019.105347.
(14) Sznajder-Katarzynska, K.; Surma, M.; Cieslik, E.; Wiczkowski, W. The perfluoroalkyl substances (PFASs) contamination of fruits and vegetables. *Food Addit Contam Part A Chem Anal Control Expo Risk Assess* **2018**, *35* (9), 1776-1786. DOI: 10.1080/19440049.2018.1502477.
(15) Groffen, T.; Prinsen, E.; Devos Stoffels, O. A.; Maas, L.; Vincke, P.; Lasters, R.; Eens, M.; Bervoets, L. PFAS accumulation in several terrestrial plant and invertebrate species reveals species-specific differences. *Environ Sci Pollut Res Int* **2023**, *30* (9), 23820-23835. DOI: 10.1007/s11356-022-23799-8. D'Hollander, W.; De Bruyn, L.; Hagenaars, A.; de Voogt, P.; Bervoets, L. Characterisation of perfluorooctane sulfonate (PFOS) in a terrestrial ecosystem near a fluorochemical plant in Flanders, Belgium. *Environ Sci Pollut Res Int* **2014**, *21* (20), 11856-11866. DOI: 10.1007/s11356-013-2449-4.
(16) Blaine, A. C.; Rich, C. D.; Sedlacko, E. M.; Hundal, L. S.; Kumar, K.; Lau, C.; Mills, M. A.; Harris, K. M.; Higgins, C. P. Perfluoroalkyl acid distribution in various plant compartments of edible crops grown in biosolids-amended soils. *Environmental Science & Technology* **2014**, *48* (14), 7858-7865. DOI: 10.1021/es500016s.
(17) Lan, Z.; Yao, Y.; Xu, J.; Chen, H.; Ren, C.; Fang, X.; Zhang, K.; Jin, L.; Hua, X.; Alder, A. C.; et al. Novel and legacy per- and polyfluoroalkyl substances (PFASs) in a farmland environment: Soil distribution and biomonitoring with plant leaves and locusts. *Environmental Pollution* **2020**, *263* (Pt A), 114487. DOI: 10.1016/j.envpol.2020.114487.
(18) Sungur, Ş.; Çevik, B.; Köroğlu, M. Determination of perfluorooctanoic acid (PFOA) and perfluorooctane sulfonic acid (PFOS) contents of compost amended soils and plants grown in these soils. *International Journal of Environmental Analytical Chemistry* **2020**, *102* (8), 1926-1934. DOI: 10.1080/03067319.2020.1745200.
(19) Blaine, A. C.; Rich, C. D.; Hundal, L. S.; Lau, C.; Mills, M. A.; Harris, K. M.; Higgins, C. P. Uptake of perfluoroalkyl acids into edible crops via land applied biosolids: field and greenhouse studies. *Environmental Science & Technology* **2013**, *47* (24), 14062-14069. DOI: 10.1021/es403094q.
(20) Felizeter, S.; Jurling, H.; Kotthoff, M.; De Voogt, P.; McLachlan, M. S. Influence of soil on the uptake of perfluoroalkyl acids by lettuce: A comparison between a hydroponic study and a field study. *Chemosphere* **2020**, *260*, 127608. DOI: 10.1016/j.chemosphere.2020.127608.
(21) Kim, H.; Ekpe, O. D.; Lee, J. H.; Kim, D. H.; Oh, J. E. Field-scale evaluation of the uptake of Perfluoroalkyl substances from soil by rice in paddy fields in South Korea. *Science of the Total Environment* **2019**, *671*, 714-721. DOI: 10.1016/j.scitotenv.2019.03.240.
(22) Navarro, I.; de la Torre, A.; Sanz, P.; Porcel, M. A.; Pro, J.; Carbonell, G.; Martinez, M. L. Uptake of perfluoroalkyl substances and halogenated flame retardants by crop plants grown in biosolids-amended soils. *Environmental Research* **2017**, *152*, 199-206. DOI: 10.1016/j.envres.2016.10.018.
(23) Wen, B.; Li, L.; Zhang, H.; Ma, Y.; Shan, X. Q.; Zhang, S. Field study on the uptake and translocation of perfluoroalkyl acids (PFAAs) by wheat (Triticum aestivum L.) grown in biosolids-amended soils. *Environmental Pollution* **2014**, *184*, 547-554. DOI: 10.1016/j.envpol.2013.09.040.
(24) Innovation, H. *Australian Horticulture Statistics Handbook 2020/21*; 2021. https://www.horticulture.com.au/growers/help-your-business-grow/research-reports-publications-fact-sheets-and-more/australian-horticulture-statistics-handbook/ (accessed 7/7/22).
(25) USEPA. Update for Chapter 9 of the Exposure Factors Handbook. National Center for Environmental Assessment: Office of Research and Development, Washington D.C., 2018.
(26) Mohidem, N. A.; Hashim, N.; Shamsudin, R.; Che Man, H. Rice for food security: Revisiting its production, diversity, rice milling process and nutrient content. *Agriculture* **2022**, *12* (6), 741.

(27) Reynolds, M. P.; Braun, H.-J. *Wheat improvement: food security in a changing climate*; Springer Nature, 2022.
(28) Australian Bureau of Statistics. *Apparent Consumption of Selected Foodstuffs*. ABS, 2021. https://www.abs.gov.au/statistics/health/health-conditions-and-risks/apparent-consumption-selected-foodstuffs-australia/2020-21 (accessed 2023 29/6/23).
(29) Schrenk, D.; Bignami, M.; Bodin, L.; Chipman, J. K.; del Mazo, J.; Grasl-Kraupp, B.; Hogstrand, C.; Hoogenboom, L.; Leblanc, J. C.; Nebbia, C. S.; et al. Risk to human health related to the presence of perfluoroalkyl substances in food. *EFSA Journal* **2020**, *18* (9), e06223.
(30) Ali, A. M.; Sanden, M.; Higgins, C. P.; Hale, S. E.; Alarif, W. M.; Al-Lihaibi, S. S.; Raeder, E. M.; Langberg, H. A.; Kallenborn, R. Legacy and emerging per- and polyfluorinated alkyl substances (PFASs) in sediment and edible fish from the Eastern Red Sea. *Environmental Pollution* **2021**, *280*, 116935. DOI: 10.1016/j.envpol.2021.116935. Ruffle, B.; Vedagiri, U.; Bogdan, D.; Maier, M.; Schwach, C.; Murphy-Hagan, C. Perfluoroalkyl Substances in US market basket fish and shellfish. *Environmental Research* **2020**, *190*, 109932. Young, W.; Wiggins, S.; Limm, W.; Fisher, C. M.; DeJager, L.; Genualdi, S. Analysis of Per- and Poly(fluoroalkyl) Substances (PFASs) in Highly Consumed Seafood Products from U.S. Markets. *J Agric Food Chem* **2022**, *70* (42), 13545-13553. DOI: 10.1021/acs.jafc.2c04673.
(31) Vestergren, R.; Orata, F.; Berger, U.; Cousins, I. T. Bioaccumulation of perfluoroalkyl acids in dairy cows in a naturally contaminated environment. *Environ Sci Pollut Res Int* **2013**, *20* (11), 7959-7969. DOI: 10.1007/s11356-013-1722-x.
(32) Gazzotti, T.; Sirri, F.; Ghelli, E.; Zironi, E.; Zampiga, M.; Pagliuca, G. Perfluoroalkyl contaminants in eggs from backyard chickens reared in Italy. *Food Chemistry* **2021**, *362*, 130178. Zafeiraki, E.; Costopoulou, D.; Vassiliadou, I.; Leondiadis, L.; Dassenakis, E.; Hoogenboom, R. L.; van Leeuwen, S. P. Perfluoroalkylated substances (PFASs) in home and commercially produced chicken eggs from the Netherlands and Greece. *Chemosphere* **2016**, *144*, 2106-2112.
(33) Macheka, L. R.; Olowoyo, J. O.; Mugivhisa, L. L.; Abafe, O. A. Determination and assessment of human dietary intake of per and polyfluoroalkyl substances in retail dairy milk and infant formula from South Africa. *Sci Total Environ* **2021**, *755* (Pt 2), 142697. DOI: 10.1016/j.scitotenv.2020.142697. Sznajder-Katarzyńska, K.; Surma, M.; Wiczkowski, W.; Cieślik, E. The perfluoroalkyl substance (PFAS) contamination level in milk and milk products in Poland. *International Dairy Journal* **2019**, *96*, 73-84.
(34) Smalling, K. L.; Romanok, K. M.; Bradley, P. M.; Morriss, M. C.; Gray, J. L.; Kanagy, L. K.; Gordon, S. E.; Williams, B. M.; Breitmeyer, S. E.; Jones, D. K. Per-and polyfluoroalkyl substances (PFAS) in United States tapwater: Comparison of underserved private-well and public-supply exposures and associated health implications. *Environment International* **2023**, 108033.
(35) Juhasz, A. L.; Keith, A.; Jones, R.; Kastury, F. Impact of precursors and bioaccessibility on childhood PFAS exposure from house dust. *Science of The Total Environment* **2023**, *889*, 164306. DOI: https://doi.org/10.1016/j.scitotenv.2023.164306. de la Torre, A.; Navarro, I.; Sanz, P.; Martinez, M. L. A. Occurrence and human exposure assessment of perfluorinated substances in house dust from three European countries. *Science of the Total Environment* **2019**, *685*, 308-314. DOI: 10.1016/j.scitotenv.2019.05.463.
(36) Shoeib, M.; Harner, T.; M. Webster, G.; Lee, S. C. Indoor sources of poly-and perfluorinated compounds (PFCS) in Vancouver, Canada: implications for human exposure. *Environmental science & technology* **2011**, *45* (19), 7999-8005.
(37) Poothong, S.; Papadopoulou, E.; Padilla-Sanchez, J. A.; Thomsen, C.; Haug, L. S. Multiple pathways of human exposure to poly- and perfluoroalkyl substances (PFASs): From external exposure to human blood. *Environment International* **2020**, *134*, 105244. DOI: 10.1016/j.envint.2019.105244.

(38) Song, Y.; Wang, Y.; Mao, W.; Sui, H.; Yong, L.; Yang, D.; Jiang, D.; Zhang, L.; Gong, Y. Dietary cadmium exposure assessment among the Chinese population. *PloS one* **2017**, *12* (5), e0177978.
(39) Liu, L.; Qu, Y.; Huang, J.; Weber, R. Per-and polyfluoroalkyl substances (PFASs) in Chinese drinking water: risk assessment and geographical distribution. *Environmental Sciences Europe* **2021**, *33* (1), 1-12.
(40) USEPA. IRIS Toxicological Review of Perfluorobutanoic Acid (PFBA, CASRN 375-22-4) and Related Salts. Development, O. o. R. a., Ed.; U.S. Environmental Protection Agency: Washington, DC, 2022.
(41) FSANZ. *Perfluorinated chemicals in food*; 2017. https://www1.health.gov.au/internet/main/publishing.nsf/Content/ohp-pfas-hbgv.htm (accessed 25 March 2021).
(42) USEPA. Drinking Water Health Advisory for Perfluorooctanoic Acid (PFOA). Agency, O. o. W. U. S. E. P., Ed.; Washington DC, 2016. USEPA. Drinking Water Health Advisory for Perfluorooctane Sulfonate (PFOS). Agency, O. o. W. U. S. E. P., Ed.; Washington DC, 2016.
(43) ATSDR. Toxicological Profile for Perfluoroalkyls. Registry, A. f. T. S. a. D., Ed.; U.S. Department of Health and Human Services, Public Health Service: Atlanta, GA, 2021.
